# Psychological factors shaping public responses to COVID-19 digital contact tracing technologies in Germany

**DOI:** 10.1038/s41598-021-98249-5

**Published:** 2021-09-21

**Authors:** Anastasia Kozyreva, Philipp Lorenz-Spreen, Stephan Lewandowsky, Paul M. Garrett, Stefan M. Herzog, Thorsten Pachur, Ralph Hertwig

**Affiliations:** 1 Center for Adaptive Rationality, Max Planck Institute for Human Development, Berlin, Germany; 2 School of Psychological Science, University of Bristol, Bristol, UK; 3 School of Psychological Sciences, University of Western Australia, Perth, Australia; 4 Melbourne School of Psychological Sciences, University of Melbourne, Melbourne, Australia

**Keywords:** Psychology, Human behaviour

## Abstract

The COVID-19 pandemic has seen one of the first large-scale uses of digital contact tracing to track a chain of infection and contain the spread of a virus. The new technology has posed challenges both for governments aiming at high and effective uptake and for citizens weighing its benefits (e.g., protecting others’ health) against the potential risks (e.g., loss of data privacy). Our cross-sectional survey with repeated measures across four samples in Germany ($N = 4357$) focused on psychological factors contributing to the public adoption of digital contact tracing. We found that public acceptance of privacy-encroaching measures (e.g., granting the government emergency access to people’s medical records or location tracking data) decreased over the course of the pandemic. Intentions to use contact tracing apps—hypothetical ones or the Corona-Warn-App launched in Germany in June 2020—were high. Users and non-users of the Corona-Warn-App differed in their assessment of its risks and benefits, in their knowledge of the underlying technology, and in their reasons to download or not to download the app. Trust in the app’s perceived security and belief in its effectiveness emerged as psychological factors playing a key role in its adoption. We incorporate our findings into a behavioral framework for digital contact tracing and provide policy recommendations.

## Introduction

Public health interventions, economic aid, and behavioral regulations have all been enlisted to curb the damage of the COVID-19 pandemic^1,2^. Before vaccines became available, behavioral measures—restricting social mixing and public gatherings, tracing the contacts of those infected, and implementing a combination of physical distancing rules and hygiene measures^3^—were the most promising way to contain the pandemic. Technological solutions have also helped stem the spread of COVID-19^4,5^. Indeed, with the exception of the Ebola outbreak in West Africa in 2014–2016^6^, the COVID-19 pandemic has seen the first large-scale use of digital contact tracing for epidemiological purposes^7^. This study examines the psychological factors that have contributed to the adoption of tracking apps during the COVID-19 pandemic.

Smartphone tracking apps use GPS, telecommunication, or Bluetooth data to create a list of contacts with whom a user may have been co-located^5^. This contact information is stored locally on the phone or on a centralized server. If a person later tests positive for COVID-19 and shares their infection status with an app, all users in their contact list can be notified instantly, allowing them to self-isolate and get tested, thus ideally helping to slow the spread of the virus^8^.

To date, about 50 countries have introduced COVID-19 contact tracing apps; most use Bluetooth tracking technologies^9^. The Corona-Warn-App, launched in Germany in June 2020, is an open-source Bluetooth-based decentralized smartphone app (https://www.coronawarn.app/en) that aims to ease the burden of the pandemic on local public health authorities by complementing their offline contact tracing efforts. The app employs a privacy-preserving model, collecting anonymized contact data that are stored locally on the user’s smartphone. Like Spain’s Radar COVID app and the United Kingdom’s NHS COVID-19 app, the Corona-Warn-App is based on the Exposure Notification system developed by Google and Apple.

The epidemiological impact of digital contact tracing apps remains low^10^ or uncertain (although the NHS app has been reported to have a positive impact;^11^): No country has been able to prevent more widespread outbreaks without implementing harsher restrictions. Most studies agree that higher participation rates are needed to increase the efficacy of digital contact tracing. In most countries, the uptake of digital contact tracing apps has high variability (varying from 13% in India to 82% in Singapore as of April 2021, see Supplementary Fig. A1 and Table A1). Early simulation models have suggested that the pandemic can be stopped if 60% of people download the app, but that lower numbers of app users can also be effective in preventing cases and deaths^12,13^. More recent simulation studies also suggest that even levels of adoption above 20% can have a mitigating impact^14,15^; and recent evidence suggests this to be the case in the United Kingdom^11^. It is therefore crucial to understand the reasons underlying people’s decision to download and use the app. Only then will it be possible to develop measures that can help to make digital contact tracing an effective long-term epidemiological measure^16^.

**Figure 1:**
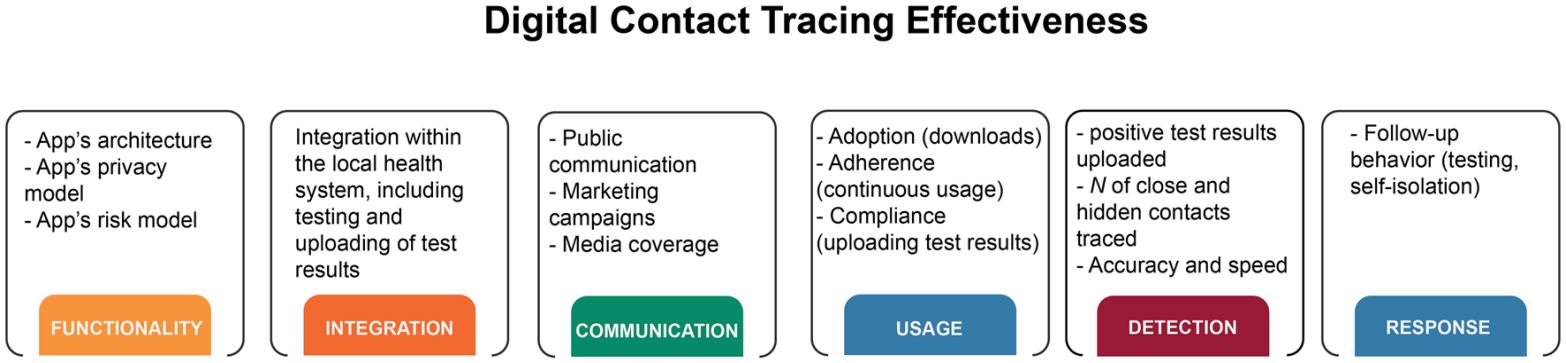
Factors contributing to the effectiveness of digital contact tracing technologies. Expanded based on the analysis by^17^.

The effectiveness of digital contact tracing technologies depends on a combination of related but distinct factors (see Fig. 1 for a graphical representation; see also Refs.^16,17^), including (1) *functionality*: the app’s architecture (e.g., which protocol or exposure notification system it uses) and its privacy and risk models; (2) *integration*: how the app is integrated into a larger environment such as the public health system and how test results are uploaded (e.g., via QR codes); (3) *communication*: including how the risks and benefits of the app are communicated to the public; (4) *usage*: including adoption of the technology (number of downloads), continuous and correct use of the app (e.g., keeping it installed and keeping Bluetooth on), and compliance (e.g., willingness and ability to upload test results); (5) *detection*: including key effectiveness metrics such as the number of positive test results uploaded as a proportion of all clinically diagnosed infections in the population, the app’s overall detection rate (i.e., the proportion of an infected person’s contacts who are notified by the app—including contacts unknown to the infected individual), and detection accuracy (i.e., the proportion of detected contacts that is free from both false positives and false negatives; see Ref.^18^); and (6) *response*: complying with risk warnings and taking appropriate measures (e.g., taking a test or self-isolating) following risk exposure notification.

Here, we focus on the adoption of digital contact tracing technologies. Numerous factors can influence people’s decision to download and use an app. For instance, a study in Germany revealed higher adoption rates of the Corona-Warn-App among respondents with a higher risk of severe illness, respondents who follow behavioral guidelines (e.g., wearing a mask), and respondents who trust the national government, the healthcare system, and science in general^19^. A study from France likewise found that higher trust in government is associated with higher acceptability and increased use of contact tracing apps^20^; similar findings have been reported for the United Kingdom^21^.

A U.S. study on the willingness to adopt warning apps has shown, using hypothetical scenarios, that people consider both the risks and the benefits of such technologies^18^. Benefits include knowing about one’s risk exposure, feeling altruistic, and protecting others; risks include privacy costs and costs of mobile data use. Another U.S. study found that people value both accuracy and privacy in a tracking app^22^. An international study likewise highlighted the importance of privacy concerns, finding that 37% of participants would not download an app even if it protected their data perfectly^23^. At the same time, studies in Australia^24^, the United Kingdom^21^, and among young adults in Taiwan^25^ have shown high acceptance of potential tracking technologies, especially in the presence of privacy-preserving conditions. Other studies and opinion pieces have highlighted the crucial role that privacy plays in public adoption of COVID-19 tracking technologies^26,27^.

**Figure 2:**
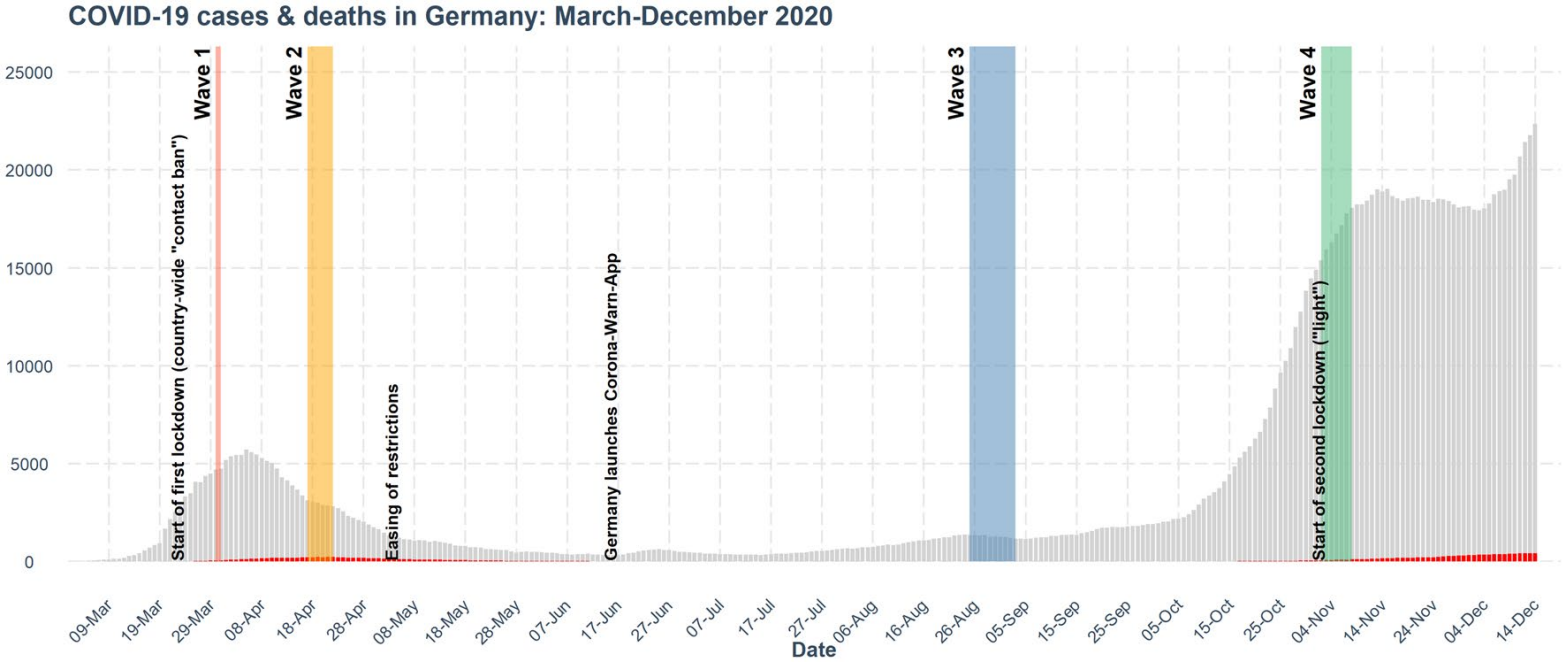
Rolling 7-day averages of daily reported COVID-19 cases (gray) and deaths (red) in Germany between March and December 2020. Data were collected at four points of measurement: Wave 1 (30–31 March), Wave 2 (17–22 April), Wave 3 (25 August–03 September), and Wave 4 (02–08 November). The timeline includes key policy decisions and the launch of the Corona-Warn-App.

This study focuses on Germany; it draws on survey data collected by an international consortium that has conducted parallel representative surveys in countries including Australia^24^, Taiwan^25^, and the United Kingdom^21^. Our study investigated two main research questions (preregistered at https://osf.io/6mkag): (1) Which factors influence the public acceptance of governmental use of location tracking data in an emergency? This includes the question of how people perceive location tracking technologies, including their data privacy and effectiveness. (2) How did people’s attitudes differ across the pandemic? Repeated measurements in cross-sectional samples allowed us to compare attitudes to hypothetical scenarios in the early waves with attitudes to and adoption of Germany’s Corona-Warn-App, which was launched after the second of our four waves of assessment (Fig. 2). Our third preregistered research question, which took a crosscultural perspective, will be addressed in a forthcoming international project report.

Our study was conducted during the first 8 months of the pandemic in Germany (March to November 2020). In each of the four waves, we examined how acceptable respondents found a range of privacy-encroaching measures (e.g., giving the government access to medical records, tracking people’s locations using mobile phone data, and temporarily relaxing data protection regulations). In the first two waves, respondents were presented with one of three hypothetical scenarios representing different degrees of privacy invasion. Each scenario described a hypothetical tracking app and the related policies (e.g., the government is required to delete all data collected by the app after 6 months). The last two waves probed respondents’ attitudes toward the Corona-Warn-App itself (see Appendix Table B4 for descriptions of the scenarios). We also collected a variety of attitude measures, such as worldviews and COVID-19 risk perceptions, in order to identify potential psychological predictors of policy acceptance (see “Methods” for details). Strengths of our approach include the ability to compare attitudes toward three hypothetical scenarios (in the earlier waves) with actual adoption rates of the Corona-Warn-App (in the later waves). Our cross-sectional study with large representative online samples thus allows us to identify the psychological factors behind the adoption of digital contact tracing technologies, and to examine how those factors change over time.

We discuss our results in the light of research on the perceived risks and benefits of new technologies and incorporate these insights into a behavioral framework for digital contact tracing (based on the behavior change wheel by Ref.^28^). Our adaptation of this framework highlights how the three components of capability, opportunity, and motivation contribute to the adoption of COVID-19 tracking technologies. We conclude by offering policy recommendations aiming to encourage the public to adopt privacy-preserving contact tracing apps.

## Results

In order to address our two main research questions of which factors influence the public response to digital contact tracing and how attitudes differ across the pandemic, we ran analyses addressing the following questions: (1) How have people’s risk perceptions of COVID-19 changed over the course of the pandemic? (2) How have people’s attitudes towards various privacy-encroaching measures changed over the course of the pandemic? (3) How acceptable do people find various types of tracking technologies? And how do these attitudes compare to download rates for the Corona-Warn-App? (4) How do people rate various measures of the effectiveness and risks of these technologies? (5) What are the most important reasons for people to download or not download the Corona-Warn-App? (6) Which factors are most predictive of app adoption and intention to download?

### COVID-19 risk perceptions

**Figure 3:**
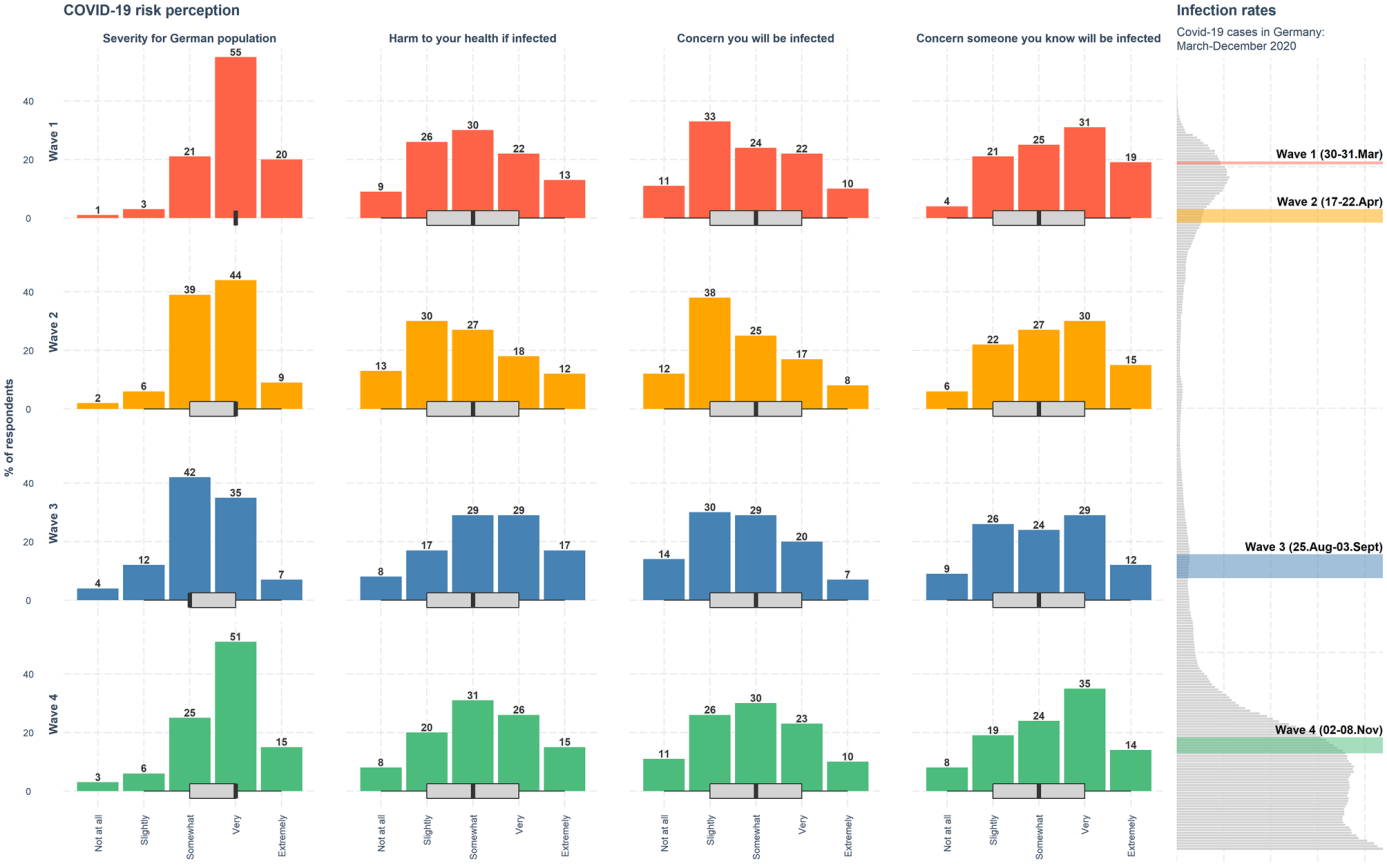
Perceived risks from COVID-19 across the four waves of the study. Barplots show the distribution of responses to the four items (with percentages). Boxplots show the interquartile range (IQR; responses between the 25th and 75th percentiles); the black line within each box indicates the median value. Lower and upper whiskers extend from the hinge to the smallest and largest values within $1.5 \times IQR$. Plot on the right shows COVID-19 infection reported in Germany over the study waves (rolling 7-day averages of daily reported COVID-19 cases). For wordings of the risk items, see Supplementary Table B1.

As Fig. 3 shows, the clear majority of participants indicated that they thought the virus posed a moderate to severe threat to the general population: The number of participants stating that the virus’s severity for the population was “somewhat,” “very,” or “extremely” high ranged from 84 to 96% across the four waves, with severity ratings increasing along with infection rates. Median values reached the “very severe” level in three of the four study waves. For all other risk perception items, median values were at the “somewhat severe” level throughout the four study waves.

The proportion of people who believed that the virus threatened their health “somewhat” remained stable (between 27 and 31%), while the proportion of people who thought the threat was “very” or “extremely” high fluctuated, tending toward higher numbers with time (March: 35%, April: 30%, September: 46%, November: 41%). Overall, on the aggregate level, people were more concerned about the health of others than about their own health as indicated by the distribution of responses in the barplots (Fig. 3).

Across all four waves, on the individual level (within respondents), the majority of participants were equally concerned about the risk of infection to themselves and to others (Supplementary Fig. A2). However, over time, people showed increased concern for themselves, reflected in the rising proportion of people concerned equally for themselves and others (March and April: 49%, September and November: 59%) and the decreasing proportion of people reporting more concern for others (March and April: 43–44%, September and November: 33%). The proportions of respondents who indicated more concern for themselves than for others remained stable (7–8%) across all four waves.

### Acceptability of privacy-encroaching measures

**Figure 4:**
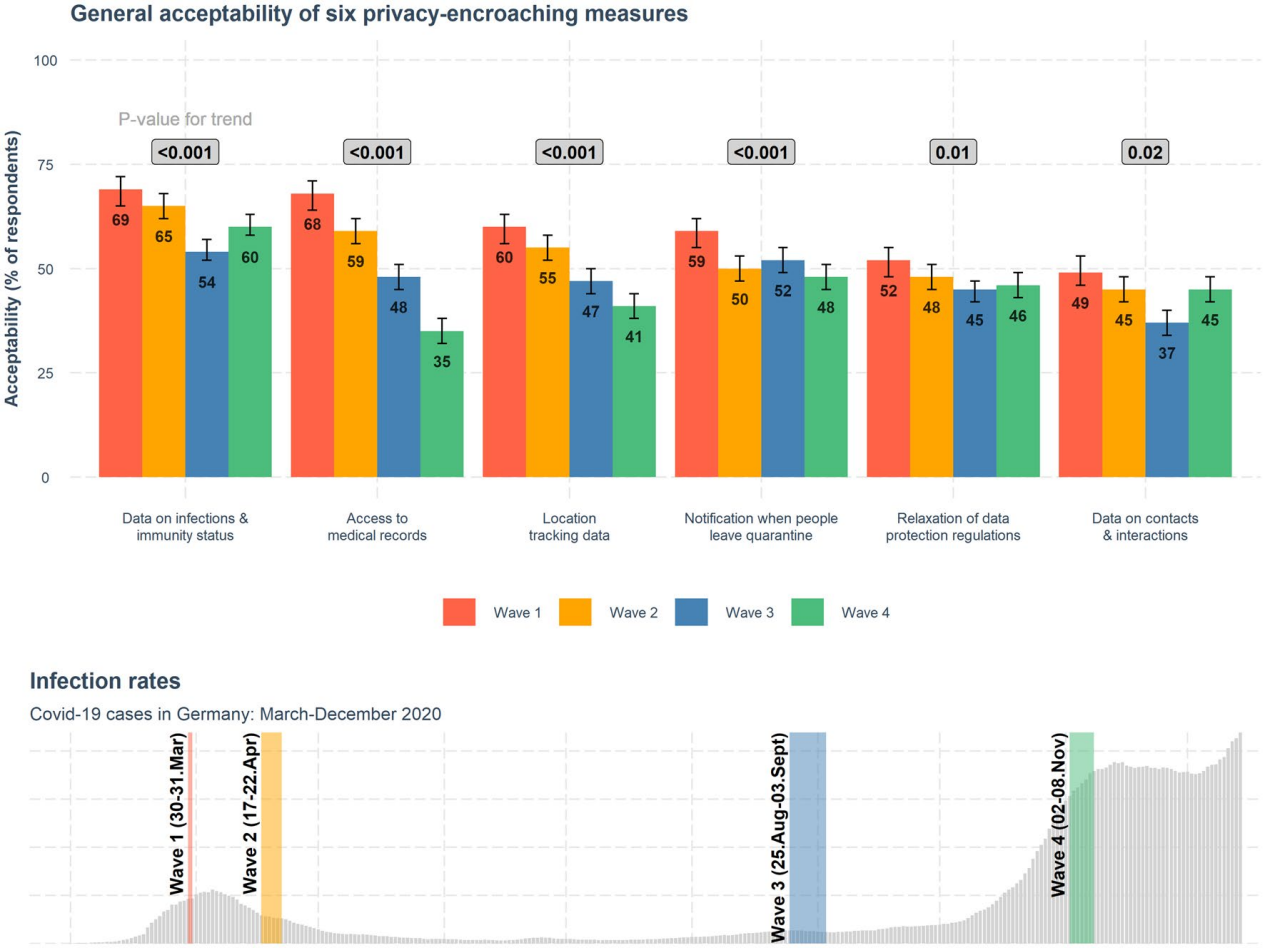
Acceptability of privacy-encroaching measures in Germany across the four waves of the study. Acceptability scores represent the total percentage of participants who chose the response options “very acceptable” or “somewhat acceptable” to the question “How acceptable is it for the government to take the following measures to limit the spread of the virus during the COVID-19 pandemic?” Error bars are 95% confidence intervals computed with the R function *prop.test*; results of this test are reported in Supplementary Table A3. *p* values from Cochran-Armitage tests for trend shown in gray boxes above the barplots; see Supplementary Table A2 for detailed results. Plot at the bottom shows COVID-19 infection reported in Germany over the study waves (rolling 7-day averages of daily reported COVID-19 cases). See Supplementary Table B9 for the wording of the items.

As Fig. 4 shows, the acceptability of privacy-encroaching measures that could hypothetically be implemented by the government (e.g., temporarily suspending data protection regulations) was fairly high, but tended to decrease over time.

Whereas respondents’ risk perceptions tracked the pandemic’s development in Germany—that is, perceived risk for the country as a whole was higher at the end of March and April (Waves 1 and 2, respectively) and November (Wave 4), when infections were rising (Fig. 3)—respondents’ attitudes toward privacy-encroaching measures followed a different pattern. After the initial shock of the pandemic, the acceptability of all measures tended to decrease from thereon, as reflected in the decreasing percentages of people who deemed these measures “very” or “somewhat” acceptable. Cochran-Armitage tests for trend corroborate the decreasing rates of acceptability across four study waves, especially for the first four measures in Fig. 4 ($p < 0.001$). For the other two measures (temporarily relaxing data protection regulations and collecting data on contacts and interactions) there was an indication of a decreasing trend ($p = 0.01$ and $p = 0.02$, respectively; see Supplementary Table A2 for detailed results).

Closer inspection revealed that, within this overall trend of decreasing acceptability over time, there were two distinct patterns of attitudinal change: a steep gradual decrease in acceptability and a pattern that more closely mirrored the development of the pandemic. Measures such as allowing access to medical records or location tracking data fall into the first pattern. Granting the government access to citizens’ medical records was deemed “very” or “somewhat” acceptable by 68% of participants in Wave 1; this number dropped in each wave, reaching just 35% in Wave 4 despite the rise in infections and new lockdown measures at that time. Acceptability of collecting people’s location tracking data followed the same pattern (Fig. 4). In contrast, measures such as collecting data on people’s infections and immunity status or their contacts and interactions seemed to be more responsive to the pandemic’s development and associated risk perceptions (see also Supplementary Fig. A3 for correlations between acceptability of privacy-encroaching measures and COVID-19 risk perceptions). For example, 49% of respondents found collecting data on people’s contacts and interactions to be “somewhat” or “very” acceptable at the end of March, during the first phase of the pandemic in Germany. This decreased over the next two waves, then rose to 45% in November, mirroring the increase in infections in Germany at that time. Note, however, that acceptance of this measure stayed below 50% across all four waves of the study.

These two distinct patterns suggest that people may have adapted their attitudes during the pandemic, distinguishing between measures they deem more appropriate (e.g., collecting data on infections and immunity status) and measures they initially deemed acceptable but whose acceptability gradually decreased over time (e.g., granting access to medical records or location tracking data).

### Acceptability of tracking technologies

**Figure 5:**
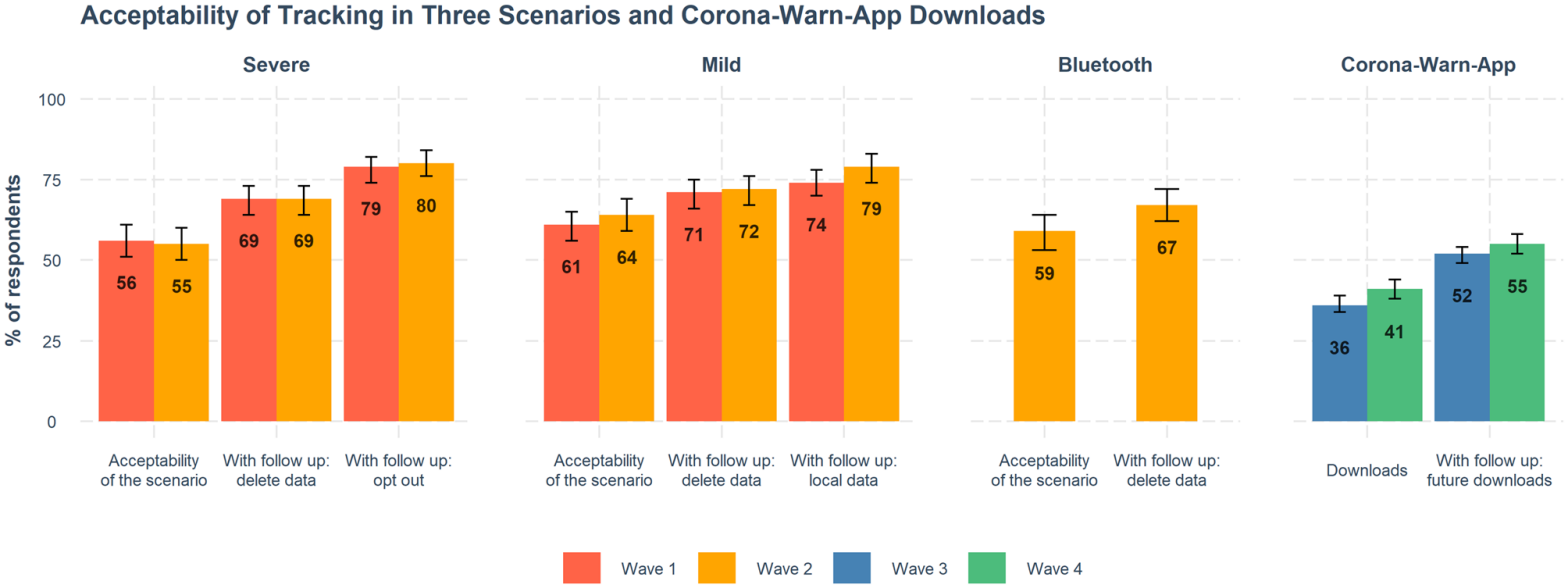
Acceptability of hypothetical tracking technologies and numbers of Corona-Warn-App downloads. For the hypothetical scenarios, the first column displays baseline acceptability ratings; the other two columns display acceptability under varying conditions: with the requirement that all data be deleted and tracking stopped after 6 months; with an “opt out” option (severe scenario); and with data being stored locally on the user’s phone (mild scenario). For Corona-Warn-App usage, the data show current downloads and intentions to download. Error bars are 95% confidence intervals computed by R function *prop.test*. Full results are reported in Supplementary Table A4. For items, see Supplementary Table B5; for descriptions of scenarios, see Supplementary Table B4.

We found relatively high levels of acceptance of the three tracking technologies presented in the hypothetical scenarios in Waves 1 and 2 (mild, severe, and Bluetooth). Acceptability of all three was above 50% in both waves. There were no large differences between proportions of respondents who found acceptable the three hypothetical scenarios (Fig. 5).

Surprisingly, although the severe scenario was deemed least acceptable (56% in Wave 1 and 55% in Wave 2 compared to 61% and 64%, respectively, for the mild scenario and 59% in Wave 2 for Bluetooth), its acceptance level was not particularly low. The differences between scenarios virtually disappeared when respondents considered follow-up options (e.g., deletion of all data after 6 months or opting out of data collection). The reported percentage of downloads of the Corona-Warn-App in our samples was smaller (36% and 41% in Waves 3 and 4, respectively) than the acceptability of the hypothetical scenarios. This low number of reported downloads is consistent with the actual download rates for the Corona-Warn-App in Germany (currently estimated at about 30% of the population; see Supplementary Fig. A1). The somewhat higher download rate reported here might be explained by the demographics of our sample, which was skewed toward online users aged 18 years or older. When respondents in Waves 3 and 4 were asked whether the Corona-Warn-App should be mandatory, only 29% and 30%, respectively, said yes (Supplementary Table A5). This could indicate that people were less likely to find tracking technologies acceptable at later stages of the pandemic (consistent with the trend in Fig. 4); it could also indicate a difference in participants’ perceptions of hypothetical scenarios and the actual app: The latter leaves less room for interpretation when participants answer the survey questions.

### Perceptions of effectiveness and risks of tracking technologies

**Figure 6:**
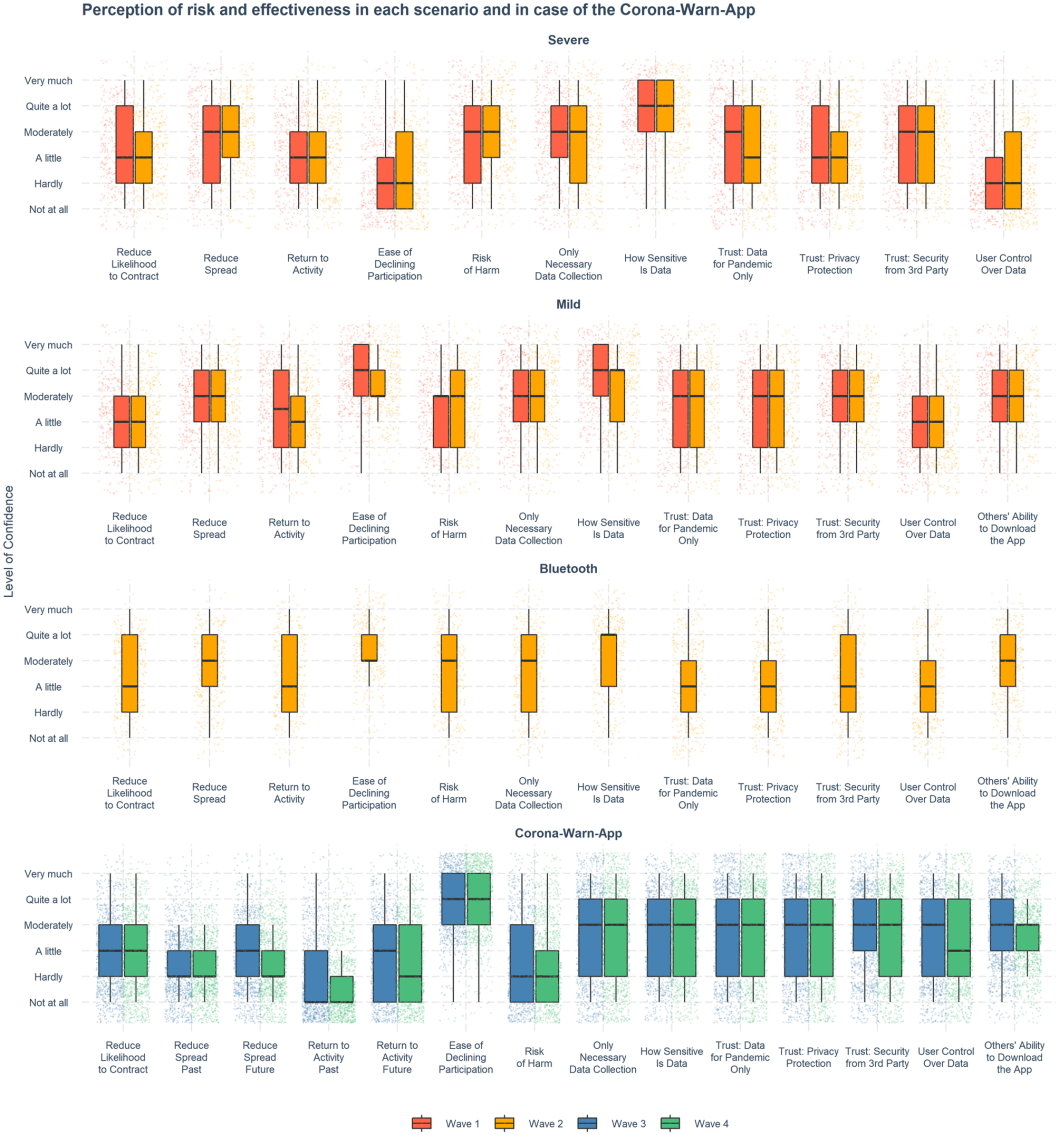
Perception of effectiveness and risks of the tracking policy in each scenario. Boxes show the interquartile range (IQR; responses between the 25th and 75th percentiles); the black horizontal line inside the boxes indicates the median value. Lower and upper whiskers extend from the hinge to the smallest and largest values within $1.5 \times IQR$. Individual responses are jittered horizontally and vertically. For items, see Supplementary Table B6; for descriptions of scenarios, see Supplementary Table B4.

Figure 6 displays perceptions of the effectiveness and risks of the tracking technologies and policies presented.

Participants understood that the severe scenario posed a greater risk to data privacy and data sensitivity, control over user data, and ability to decline participation than the other hypothetical scenarios. At the same time, they judged the potential effectiveness of the severe scenario to be on the same level as in the other two scenarios (mild and Bluetooth). It is therefore puzzling that the acceptability of the severe scenario was almost on par with the other two (see Fig. 5), even though participants thought the risk to privacy protection and the level of intrusion in citizens’ lives was much higher.

Figure 6 also shows that although participants thought the Corona-Warn-App presented only a low risk of harm, they were pessimistic about its effectiveness, including its ability to reduce the spread of the virus and to help people return to their normal activities. This pessimism toward the Corona-Warn-App was stronger than that toward the hypothetical scenarios presented in earlier waves of the study. Moreover, participants showed only moderate levels of trust in the Corona-Warn-App’s security (including trust in collection of only necessary data and only for the purposes related to the pandemic, trust in privacy protection and trust in data security from third parties)—closest to that found in the mild scenario, but higher than that in the severe and Bluetooth scenarios. Given that the technology in the Bluetooth scenario was attributed to Apple and Google, while the Corona-Warn-App was released by the German government, the lower level of trust in the Bluetooth scenario may be due to a lack of trust in commercial corporations and their standards of data protection.

**Figure 7:**
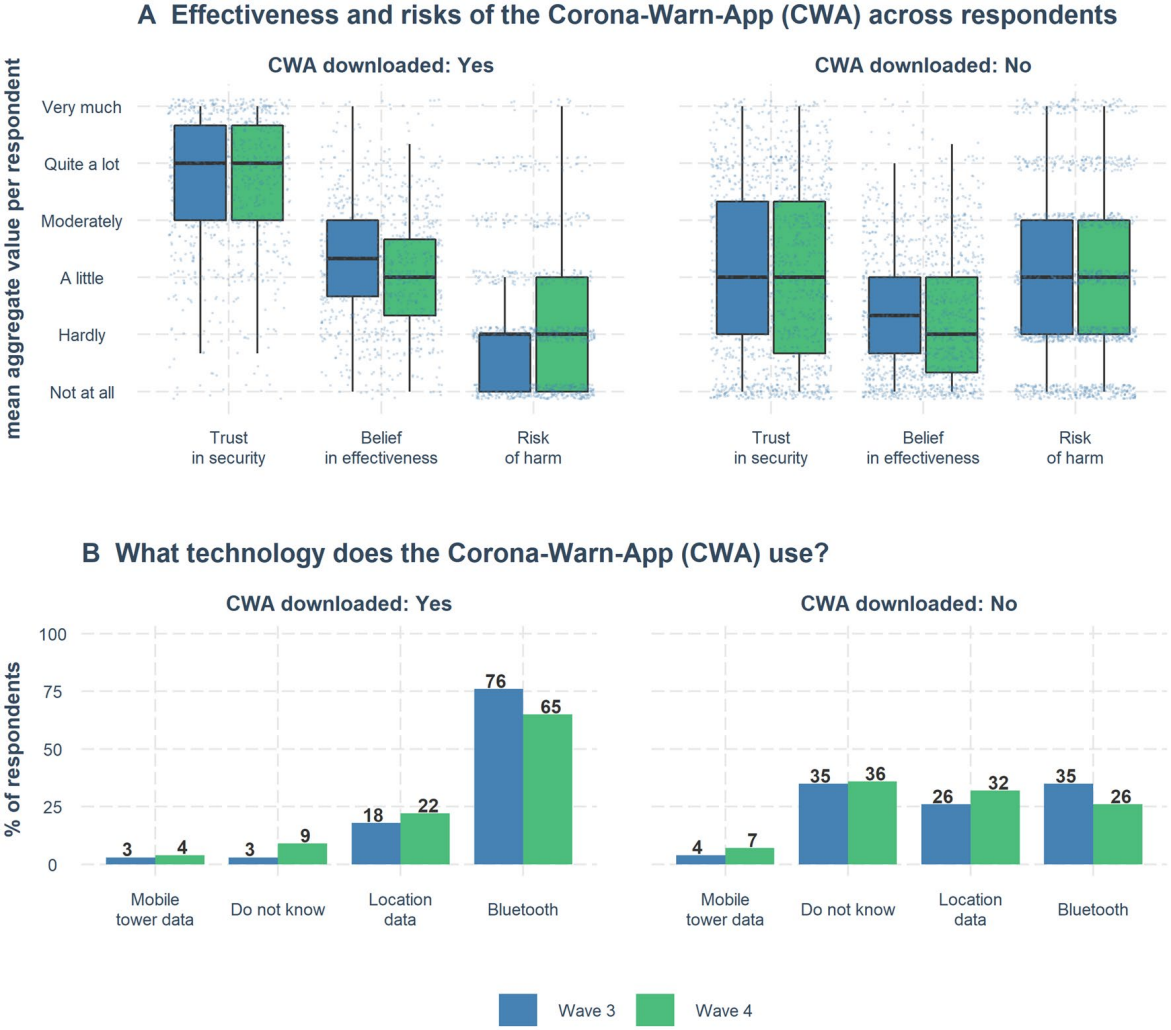
Users and non-users of the Corona-Warn-App: Perception of effectiveness, risks, and understanding of technology. (**A**) Perception of effectiveness and risks of the Corona-Warn-App for two groups: those who had downloaded the app and those who had not. The variables “Trust in security” and “Belief in effectiveness” represent combined and averaged measures from variables presented in Fig. 6. The variable “Trust in security” combines four variables: “Only Necessary Data Collection,” “Trust: Privacy Protection,” “Trust: Security from 3rd Party.” The variable “Belief in effectiveness” combines three variables: “Reduce Likelihood to Contract,” “Reduce Spread Future,” “Return to Activity Future.” “Risk of harm” is a single variable. For items, see Appendix Tables B6 and for all aggregate measures see B10. Boxplots show distributions of aggregate (mean) values. Boxes show the interquartile range (IQR; responses between the 25th and 75th percentiles); the black horizontal line inside the boxes indicates the median value. Lower and upper whiskers extend from the hinge to the smallest and largest values within $1.5 \times IQR$. Individual responses are jittered horizontally and vertically. (**B**) Understanding of the Corona-Warn-App technology. Participants who had downloaded the app were much more likely to give the correct answer: Bluetooth.

People’s perceptions of the risks and benefits of the Corona-Warn-App differed depending on whether or not they already had downloaded the app. On the aggregate level, app users judged its risk of harm to be very low and trusted the app’s security a lot (see top part of Fig. 7A). In contrast, non-users’ aggregate ratings of both risk of harm and trust in security were both at the same level (median = “a little”). This pattern was also present on the individual level (within respondents), where, for instance, the majority of users (86% and 82% in Waves 3 and 4, respectively) rated the app’s security higher than its risk of harm. At the same time, only about half (50% and 51%) of non-users rated the app’s security higher than its risk of harm, and even fewer non-users (38% and 39%) rated its effectiveness higher than its risk of harm. The opposite was true for app users, among whom the majority (77% and 70%) rated the app’s effectiveness higher than its risk of harm (see Supplementary Fig. A4).

Knowledge of the technology used by the Corona-Warn-App also differed depending on whether or not respondents had downloaded it. As Fig. 7B shows, many non-users did not understand how the Corona-Warn-App works: only 35% (Wave 3) and 26% (Wave 4) of them knew that it uses Bluetooth technology—relative to 76% (Wave 3) and 65% (Wave 4) of respondents who had downloaded the app.

To delve deeper into these differences and their impact on people’s adoption of digital contact tracing technologies, in our follow-up questions and analyses we explored potential drivers of the decision to download or not download the Corona-Warn-App.

### Corona-Warn-App: reasons for and against download

**Figure 8:**
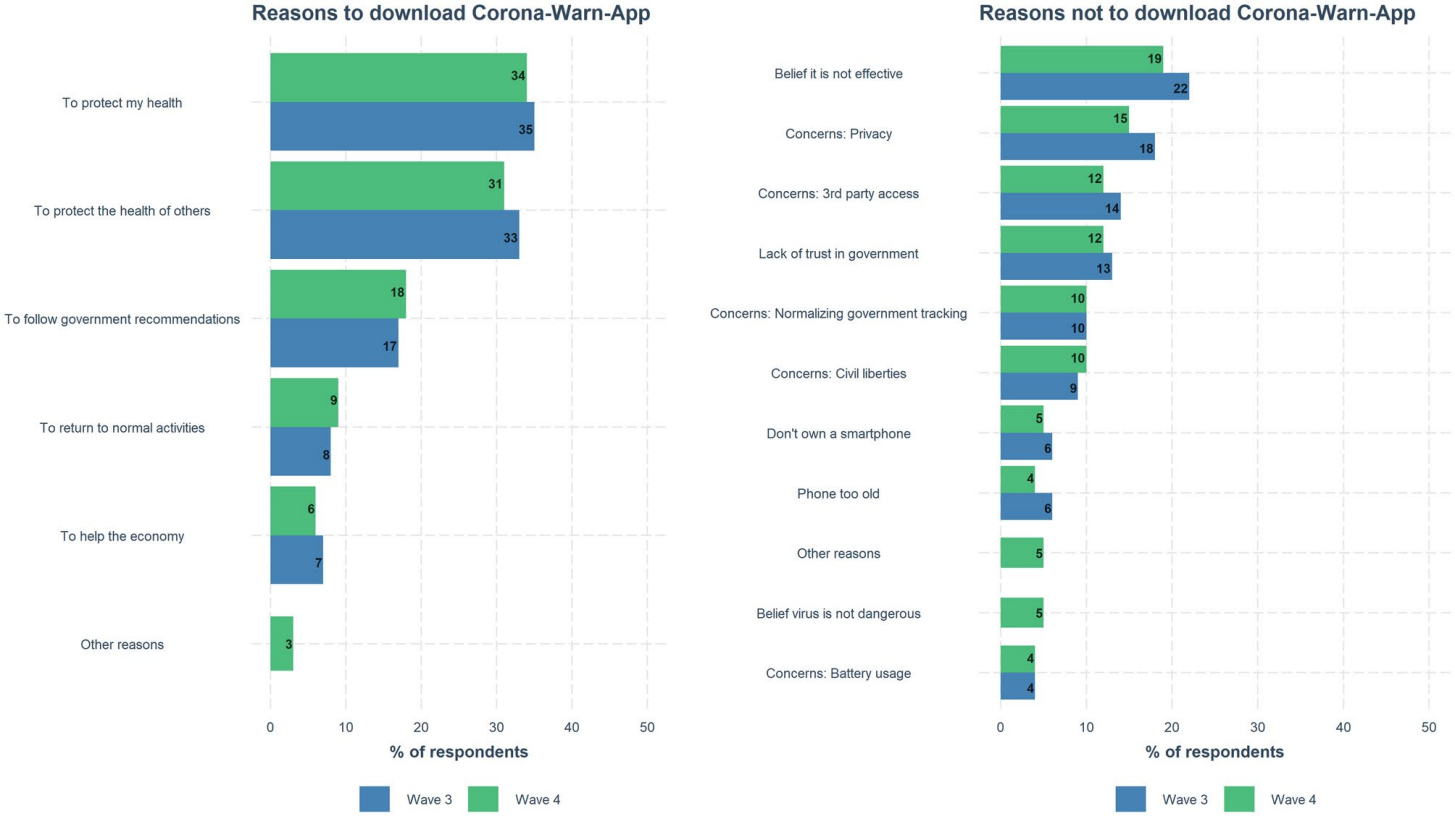
Self-reported reasons to download or not download the Corona-Warn-App. Panels show results of multiple-choice items in Waves 3 and 4. By design, there are more response options for both questions in Wave 4 than in Wave 3. See Supplementary Table B7 for the wording of the items.

The relatively low uptake of the Corona-Warn-App could be due to a variety of factors. To explore these factors, we presented respondents with several possible reasons to download or not download the Corona-Warn-App (multiple selections allowed; Fig. 8).

The results indicate that people’s main reason for downloading the app was their desire to protect their health and the health of others. The two leading reasons for people not downloading the app were the belief that the app is not effective and privacy concerns. Concerns about third-party access and lack of trust in the government also played a role.

**Figure 9:**
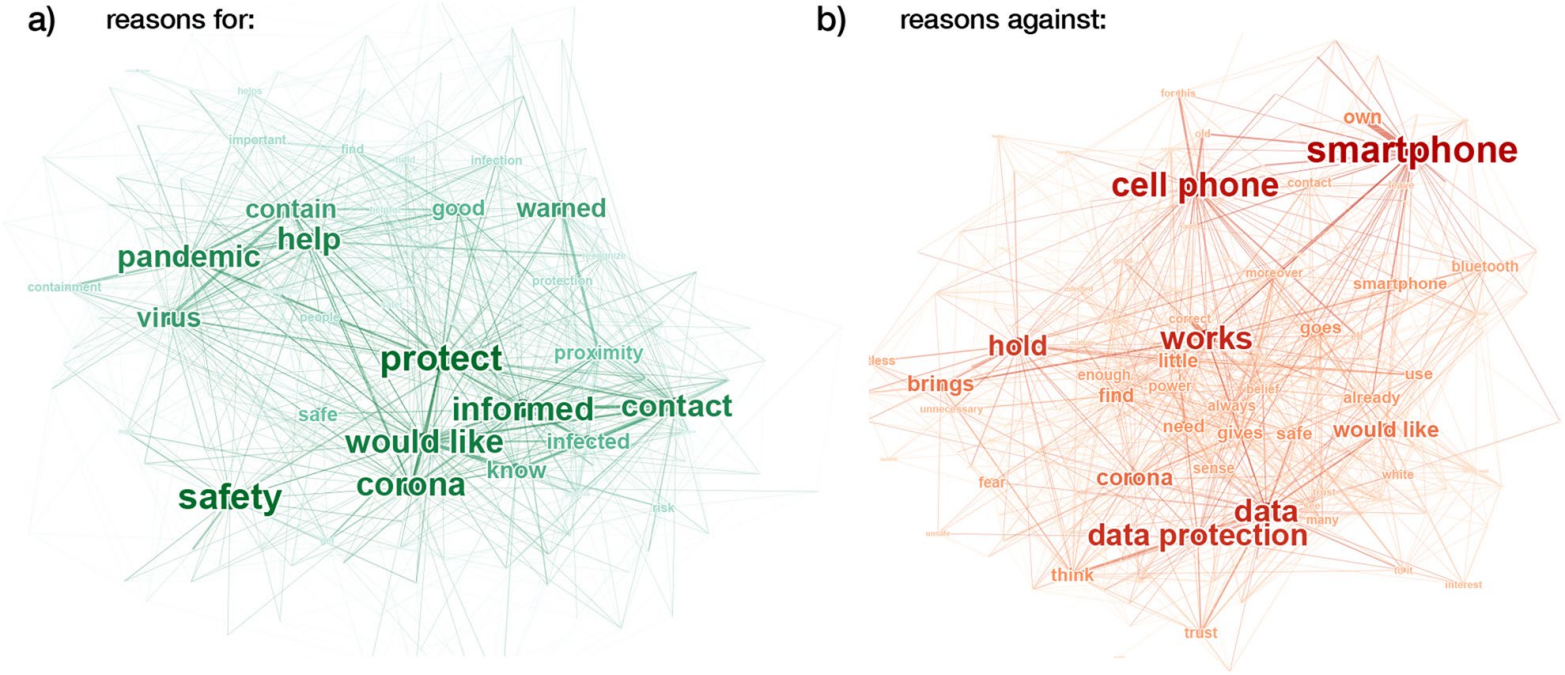
Self-reported reasons to download or not download the Corona-Warn-App in an open-response question (Wave 4 only; reasons for: *N* = 477; reasons against: *N* = 530). Co-occurrence networks of unigrams from reasons for (**a**) and against (**b**) downloading the app. Connections appear whenever two words were used by the same participant. Node and font sizes and color code are proportional to the absolute frequency of the corresponding word; nodes are positioned using a spring layout. Only unigrams that appeared at least three times in the responses are shown. Translation on a unigram basis via DeepL.com; visualization via Gephi^29^.

To analyze responses to the open-ended question about reasons to download or not download the app, we extracted unigrams (i.e., individual words) from the responses and counted their overall frequencies as well as their co-occurrences within responses. Figure 9 shows the resulting co-occurrence networks for reasons to download the app from 477 individual responses (panel a) and reasons to not download the app from 530 individual responses (panel b). Clusters of frequent words indicate the main arguments.

Reasons for downloading the app include protecting oneself and others (around the term “protect”), being informed about infections in one’s environment (“informed” and “contact”), and helping to mitigate the pandemic (“pandemic,” “contain,” and “help”). Reasons against downloading the app include technical issues (“smartphone”), data privacy (“data protection” and “trust”), problems of functionality (“works”), and doubts around how useful and necessary the app is (“hold” and “brings [nothing]”, translated from German where these words are part of expressions that mean “I consider” and “pointless”). Overall, the reasons for not downloading the app are slightly more diverse than the reasons for downloading it; this was also the case for the multiple-choice question (Fig. 8). The main difference between the multiple-choice and the open-ended responses is the more prominent role of problems with smartphones (reasons against) and of being informed (reasons for) in the open responses.

### Corona-Warn-App: predictors of download

**Figure 10:**
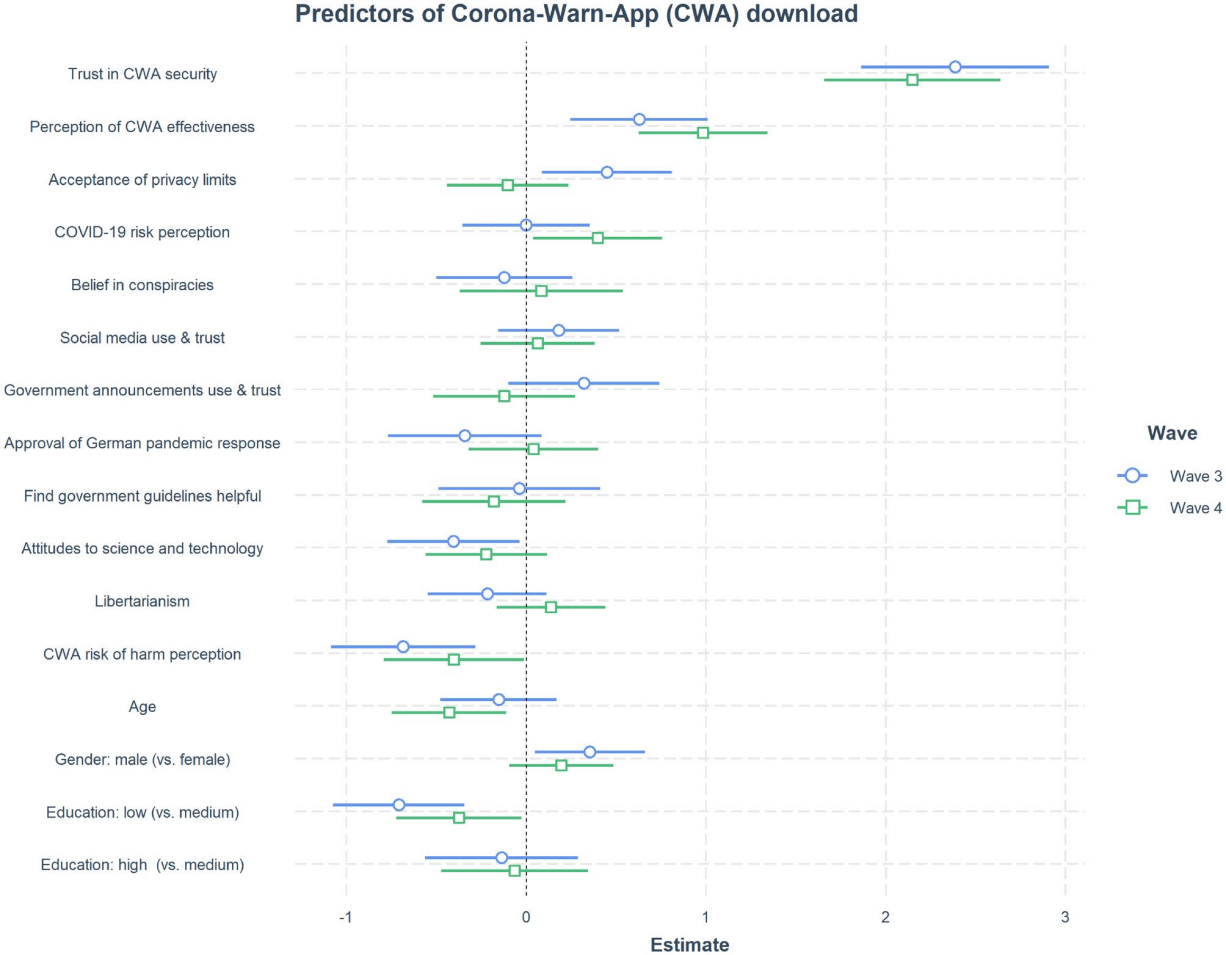
Logistic regression models predicting Corona-Warn-App download in Waves 3 and 4. Dependent variable: downloaded the app (yes/no). Coefficients: measures from the survey (e.g., combined score for trust in CWA security; combined score for conspiracy beliefs; see Supplementary Table B10). Horizontal point ranges show point estimates and 95% confidence intervals for each predictor. Education was dummy coded with the reference level “medium,” yielding two coefficients: low (vs. medium) and high (vs. medium) education. Following^30^, we standardized all continuous variables by two standard deviations (SD) and mean centered the binary gender variable. This way a 2-SD change in a continuous predictor variable is approximately equivalent to a change of category in a roughly balanced binary predictor variable (e.g., gender). In a logistic regression model, a slope reflects the relative change in log odds (while keeping all other predictors at their average values). Supplementary Table A6 summarizes the regression results for these two models. Supplementary Figures A6 and A7 display Pearson correlations for all variables in the regression model. Supplementary Figure A8 provides an alternative arrangement of the same results, where the respective model for each wave is shown in a separate panel.

To further examine why people chose to download the Corona-Warn-App, we fitted a logistic regression model using a set of independent variables measured in the survey as predictors for the dependent variable of downloading the Corona-Warn-App (Fig. 10). Once again, trust in the app’s security and perceived effectiveness emerged as leading positive predictors of downloading the app. These two variables represent combined measures made up of variables presented in Fig. 6. The variable “Trust in CWA security” included items asking respondents how much they trusted the app to ensure individuals’ privacy (“Trust: Privacy Protection”) and to only collect and use the Corona-Warn-App data to deal with the pandemic (“Only Necessary Data Collection” and “Trust: Data for Pandemic Only”), as well as how secure they thought the data collected by the app actually is (“Trust: Security from 3rd Party’)’ . The variable “Perception of CWA effectiveness” included items asking for people’s assessment of whether the app will help reduce the virus’s spread (“Reduce Spread Future”), reduce their likelihood of coming into contact with the virus (“Reduce Likelihood to Contract”), and help them return to their normal activities (“Return to Activity Future”). This variable therefore represents people’s assessment of the app’s potential to impact the course of the pandemic and help them personally (see Supplementary Table B10 for all predictor variables).

As trust in the app’s security and perceived effectiveness emerged as strong predictors of having downloaded the Corona-Warn-App, we used the same modeling approach to assess predictors of both of these variables separately. Supplementary Figures A10 and A11 show the results of the respective linear regression models. Acceptance of privacy limits during the pandemic (a combined measure summarizing all six items discussed under “Acceptability of Privacy-Encroaching Measures” and in Fig. 4) and trust in science and government guidelines emerged as moderate positive predictors of both variables. Furthermore, believing in conspiracy narratives and perceiving the Corona-Warn-App as harmful were associated with lower trust in the app’s security, but not with the perception of its effectiveness. Demographic factors such as a high level of education and identifying as male—but not age—also emerged as positive predictors of having downloaded the Corona-Warn-App. As Supplementary Fig. A5 shows, proportions of respondents who reported having downloaded the app were higher at high and medium levels of education than at a low level: For instance, 46% of participants with a university degree had downloaded the app, relative to just 29% (Wave 3) and 35% (Wave 4) of participants with a low level of education. Slightly more male respondents (Wave 3: 44%, Wave 4: 40%) than female respondents (Wave 3: 38%, Wave 4: 32%) reported having downloaded the Corona-Warn-App.

**Figure 11:**
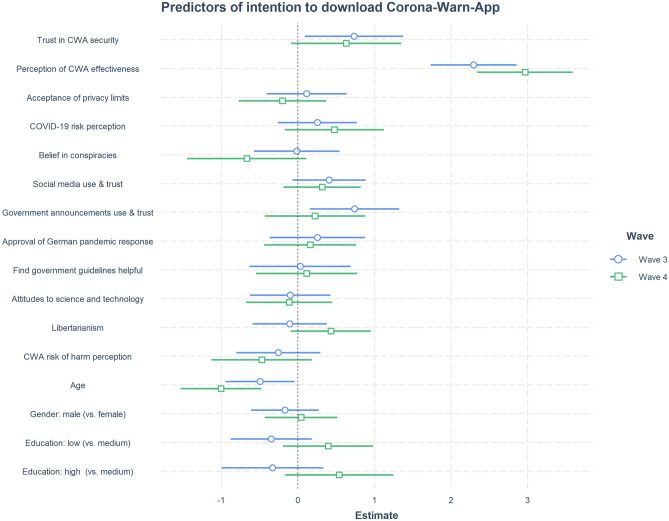
Logistic regression models for intention to download the Corona-Warn-App (Waves 3 and 4). Dependent variable: intention to download the app in the future (yes/no). Coefficients: measures from the survey (e.g., combined score for trust in app security; combined score for conspiracy beliefs; see Supplementary Table B10). Horizontal point ranges indicate point estimates and 95% confidence intervals for each predictor. Education was dummy coded with the reference level “medium,” yielding two coefficients: low (vs. medium) and high (vs. medium) education. Following^30^, we standardized all continuous variables by two standard deviations (SD) and mean centered the binary gender variable. This way a 2-SD change in a continuous predictor variable is approximately equivalent to a change of category in a roughly balanced binary predictor variable (e.g., gender). In a logistic regression model, a slope reflects the relative change in log odds (while keeping all other predictors at their average values). Supplementary Table A7 summarizes the regression results for both models. Supplementary Figure A9 provides an alternative arrangement of the same results, where the respective model for each wave is shown in a separate panel.

Finally, we used logistic regression models to analyze the intention to download the Corona-Warn-App among respondents who reported that they had not already done so. Perceived effectiveness of the app emerged as the strongest predictor of intention to download (Fig. 11) in both Waves 3 and 4. This finding is in line with self-reported reasons for not downloading the app, where effectiveness concerns (along with privacy concerns) played a large role (see Fig. 8).

## Discussion

### Which psychological factors contribute to public adoption of digital contact tracing technologies?

Digital contact tracing apps have been introduced in many countries to help to contain COVID-19 outbreaks. The apps were launched with high hopes, but have also posed a number of challenges both for governments aiming at high and effective uptake and for citizens weighing the benefits (e.g., public and individual health) against the potential risks (e.g., loss of data privacy) of these unprecedented measures. Although their epidemiological impact is presently limited or uncertain (Ref.^10^; but see Ref.^11^), digital contact tracing apps are poised to become an established epidemiological tool. Ideally, along with other measures, they can help to prevent large outbreaks by relieving the burden on public health agencies. With this potential in mind, it is crucial to understand which factors contribute to the public uptake of digital contact tracing apps. In the present survey in Germany, we focused on psychological factors that foster or inhibit the public adoption of digital contact tracing technologies.

Research on risk perception has identified several psychological drivers that figure in people’s risk–benefit calculations and contribute to the acceptance of novel technologies (see Refs.^31–34^). A first factor is the *subjective perception of risks*, including a risk’s *severity*, defined in terms of perceived lack of control and catastrophic potential (“dread risks”), and *novelty*, defined in terms of unobservable and unpredictable consequences and their potential harm (“unknown risks”; see Ref.^31^). A second factor is the *perception of benefits*, both personal (e.g., returning to normal activities, avoiding infection) and public (curbing the virus’s spread; see also Refs.^32^). A third and related factor is individuals’ *degree of knowledge and experience* of a technology, which facilitates or precludes accurate assessment of its risks and benefits (e.g., Ref.^35^, see also Ref.^36^). A fourth factor is *trust* in the authorities that develop, deploy, or supervise the technology. The trust factor becomes particularly important in the absence of knowledge about technology, as a way to cope with the complexity of the task by relying on experts or relevant authorities for risk management^33,36^. An additional factor to consider in risk–benefit assessments of digital contact tracing technologies is the context (e.g., the risk posed by the pandemic itself) and its development (e.g., whether infection rates are decreasing or increasing). For example, people might be willing to overlook risks to their privacy temporarily in order to mitigate the health risks posed by the pandemic. Results of our study speak to several of these factors, as summarized in the following sections.

#### Privacy in the context of COVID-19

We found that public acceptance of potential privacy-encroaching measures in the context of the pandemic decreased over time. This trend was especially pronounced for such hypothetical measures as granting the government emergency access to people’s location tracking data and medical records (see Fig. 4). Acceptability of all six hypothetical privacy-encroaching measures across the four waves was correlated with people’s COVID-19 risk perceptions (see Supplementary Fig. A3). Moreover, our results suggest that people might distinguish between measures they considered to be more appropriate during the pandemic (e.g., collecting data on infections and immunity status) and measures that were initially deemed acceptable but whose acceptability gradually decreased over time (e.g., granting access to medical records or location tracking data).

#### Severity and acceptability of digital tracking

Acceptability ratings for all three hypothetical scenarios in Waves 1 and 2 were high, as were intentions to download the Corona-Warn-App in Waves 3 and 4. Surprisingly, the details of the tracking technologies presented in the scenarios made little difference to public acceptance: The severe scenario involved harsh, nearly oppressive measures, while the mild and Bluetooth scenarios were compatible with privacy protection standards. However, privacy measures such as having all data deleted after 6 months or having the ability to opt out increased levels of acceptance. Similarly high acceptance rates for all three scenarios have been observed in Australia^24^, the United Kingdom^21^, and Taiwan^25^.

Taken together, these findings indicate that although people might accept certain limitations to their privacy in a crisis, they are also wary of privacy-encroaching measures and weigh the potential benefits of disclosing sensitive data against the potential risks (see also Ref.^37^). Long-term tracking solutions should therefore not rely on privacy-encroaching measures. Instead, they should build on privacy-preserving technologies, which are more likely to be accepted in the long term.

#### Perceived benefits and risks, and people’s knowledge about the Corona-Warn-App

Perceived benefits, both personal and societal, played a key role in people’s self-reported reasons to download the Corona-Warn-App (Fig. 8). Although the perceived risk of harm was low overall (Fig. 6), concerns about data privacy and government tracking were high among non-users of the app and were the leading reported reasons not to download the app (Fig. 8). Users and non-users of the app also showed different patterns in their assessment of the app’s risks and benefits: Users judged the risk of harm to be very low and reported high trust in its security; non-users, in contrast, rated both risk of harm and trust in security at equally low levels (Fig. 7A). Although users were, on average, more optimistic about the app’s effectiveness than non-users, beliefs in the app’s effectiveness were in general not high—as also observed by Ref.^19^. Users and non-users also differed in their knowledge of the Corona-Warn-App: Many non-users were not aware that that it uses Bluetooth technology (Fig. 7B). The same pattern of results was observed for Australia’s COVIDSafe app^24^.

#### Trust

Our results show that trust in the security of the Corona-Warn-App app plays a crucial role in its uptake. Trust was especially important for past decisions to download the app, while belief in its effectiveness was especially important for future download intentions (see Figs. 10 and 11).The observed importance of trust is in line with the findings of other studies exploring attitudes toward and uptake of digital contact tracing technologies: Studies in the United Kingdom^21^, Germany^19^, and France^20^ all show that trust in the government is correlated with the acceptability and use of digital contact tracing apps. Trust in governments, scientists, and health authorities has also been shown to be a strong predictor of adherence to protective measures and guidelines during the pandemic (e.g., Refs.^38–40^). Likewise, trust has proved to be a strong predictor of people’s attitudes to other novel technologies, such as 5G^34^ or gene technology^33^. These converging findings support the notion that trust in relevant authorities can function as a substitute for knowledge about a technology and its risks and benefits (see Ref.^36^).

Finally, our findings indicate that pro-free market attitudes, as a proxy for conservative political views, play only a limited role in the adoption of the Corona-Warn-App (see the predictor “Libertarianism” in Figs. 10 and 11). This suggests that public health measures such as digital contact tracing technology are not, at least not entirely, evaluated through polarized, partisan perspectives. Because the libertarianism–conservatism dimension is most pronounced in the Anglosphere, and arguably less so in Germany, this finding should be treated with caution. Yet U.S. respondents’ willingness to adopt contact tracing technologies has also been shown to be unrelated to their political leanings, with both Democrats and Republicans almost equally willing to install a potential COVID-19 tracking app^41^.^42^ also found comparable levels of support for government efforts to encourage digital contact tracing among Democrats and Republicans. A similar lack of political polarization was also observed in another relatively novel technological domain, namely, people’s attitudes to personalization of online content^43^.

### A behavioral framework for digital contact tracing

**Figure 12:**
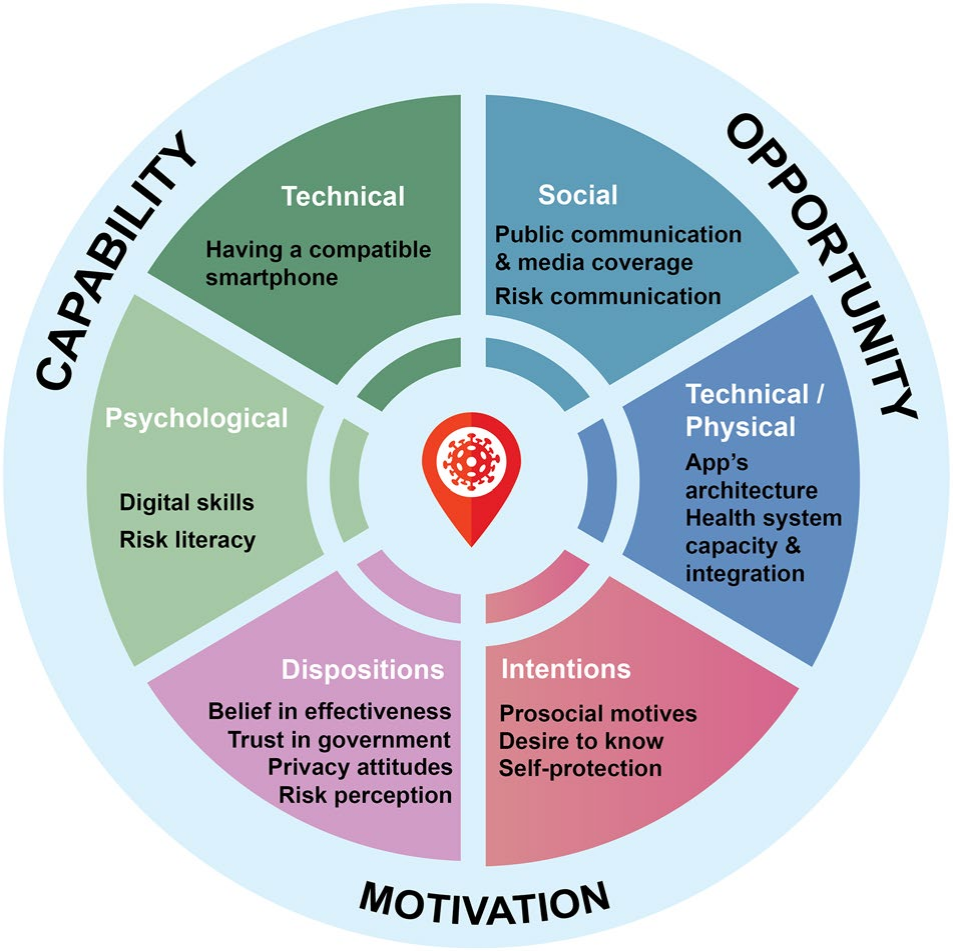
Behavioral framework for digital contact tracing. Adapted from the behavior change wheel^28^.

Our analyses highlight several factors that seem to shape people’s attitudes toward digital contact tracing technologies, including privacy concerns, trust in the app’s security, and beliefs about its effectiveness. In order to conceptualize the confluence and interplay of these factors, we suggest mapping them out within a behavior change framework^28^ such as that shown in Fig. 12. This framework consists of three components, whose interaction determines behavior: capability (an individual’s psychological and physical capacity to engage in a behavior), opportunity (environmental affordances and external factors that enable or prompt a behavior), and motivation (mental processes that guide behavior; e.g., habits, emotions, decisions^28^).

*Capability* encompasses technical capacity (i.e., having a smartphone) and the skills required to download and use the app, as well as the digital skills and risk literacy necessary to understand risk warnings in the app and to communicate test results to the app. In our samples, the majority of participants had a smartphone (Table 1), and only about 5% of participants cited not having a smartphone as a reason for not downloading the app (see Fig. 8). Nevertheless, technical problems related to smartphones (e.g., not having one or the app not working properly) played a prominent role in the open-response questions (see Fig. 9). Almost all respondents who reported having downloaded the app also reported that it was still installed on their phone (Wave 3: 92%, Wave 4: 93%) and that they kept Bluetooth switched on either always or when leaving the house (Wave 3: 95%, Wave 4: 93%; Supplementary Table A5).

*Opportunity* encompasses social and technical/physical factors external to the individual themselves. Social factors include successful communication of the app’s benefits and how to use it, as well as risk communication that explains the risk warnings and their implications for individual behavior. Technical/physical factors include the app’s architecture (e.g., where data are stored, the system’s security) and the broader system in which it is embedded—for example, the health care system. Connecting opportunity in this behavioral framework to the factors that contribute to effectiveness presented in Fig. 1, it is clear that a digital contact tracing app must be integrated into the national health care system in order to ensure ease of use (e.g., communicating a positive test result anonymously and without friction).

Decentralized privacy-respecting apps like the Corona-Warn-App represent a laudable attempt to create an opportunity to contain virus spread that respects the data minimization and protection principles set out in Article 5 of the European Union’s General Data Protection Regulation^44^. Yet clear communication of the app’s privacy model and risk model is also necessary. Given that most respondents who had not yet downloaded the app did not understand how it works (Fig. 7B), it is possible that poorly informed decision making or a knowledge gap keeps uptake unnecessarily low.

*Motivation* encompasses two key factors that are supported by our analyses of the reasons for and predictors of downloading the Corona-Warn-App. One factor comprises people’s intentions to protect themselves and others, to stay informed, and to curb the spread of the virus (Figs. 8 and 9). The other comprises people’s underlying dispositions, such as privacy attitudes, trust in government and technology, and perceptions of risks and benefits (Figs. 6 and 10). Balance between these two factors is important. For instance, even people driven by prosocial motives may decide against downloading a technology they do not trust. When people’s intentions conflict with their underlying dispositions, the resulting trade-offs may play into their decision to not adopt digital contact tracing.

Taking into account the interdependency of all these factors in a behavior system is essential not only to understanding people’s behaviors regarding digital contact tracing, but also to designing successful behavioral interventions and communication strategies.

## Conclusion and policy implications

Which insights from our study could be used to inform public policy? First, it is vital not to compromise on privacy. Although privacy-encroaching measures might initially be accepted in times of crisis, they are unlikely to be accepted on the long term. Moreover, trust in the app’s security was the leading predictor of Corona-Warn-App uptake and data privacy concerns were among the most-cited reasons for not downloading the app. Second, educate people who have not yet downloaded the app about the underlying technology, privacy model, and risk model. Third, make the app and the uploading of test results as simple and convenient as possible. Fourth, address the issue of trust—for example, by effectively demonstrating and communicating how the app preserves privacy, and clarifying that neither the government nor any other institution has access to users’ data.

To address effectiveness concerns and increase the benefits for users, more useful functions should be incorporated into digital contact tracing apps. For instance, the Corona-Warn-App now offers information about infection numbers in Germany, a digital check-in system (e.g., for shops or events) via QR codes, and the possibility to integrate digital vaccination certificates (https://www.coronawarn.app/en/blog/).

Finally, our findings suggest that arguments for digital contact tracing technologies may be particularly effective when the messaging focuses on prosocial motives, such as helping to stop the spread of the virus, and personal benefits, such as protecting one’s own health. Messaging should also address concerns about the app’s effectiveness and data security. The effectiveness of framing messages along these lines should be empirically tested.

If digital contact tracing technologies are to become a long-term solution for managing viral infectious diseases such as COVID-19, they must be effective, understandable, and acceptable to most people.

## Methods

### Participants and procedure

Four representative online samples of German participants (total retained participants $N = 4357$) were recruited through the online platform Lucid using quota sampling to account for current population distributions with regard to age (> 18 years), gender, and region (see Table 1 for information about the study, smartphone use, and basic demographics, and Fig. 2 for data collection times in relation to the pandemic’s development in Germany). Supplementary Table A10 provides additional information on educational and regional distribution for the four waves. Informed consent was obtained from all participants and the studies were conducted in accordance with relevant guidelines and regulations. The Institutional Review Board of the Max Planck Institute for Human Development approved the surveys (approval L2020-4).

**Table 1:** Study and demographic information.

	Wave 1	Wave 2	Wave 3	Wave 4
**Recruitment**				
Date of data collection	30–31.03.20	17–22.04.20	25.08–03.09.20	02–08.11.20
Sample size (recruited)	1224	1665	1633	1518
Sample size (retained)	829	1109	1231	1188
**Scenarios**				
	Severe, mild	Severe, mild, bluetooth	Corona-Warn-App	Corona-Warn-App
**Smartphone use (%)**				
No	–	3.6	7.4	6.7
Yes	–	96.4	92.6	93.3
**Gender (%)**				
Female	50.4	50.2	49.6	50.6
Male	49.2	49.3	50.1	49.3
Other	0.4	0.5	0.2	0.1
**Age (in years)**				
Median	48.0	48.0	51.0	50.0
SD	17.0	16.0	17.0	18.0

### Study design

There were four waves of data collection (for dates and sample information, see Table 1 and Fig. 2), the timing of which was determined by two main criteria: The first criterion was the development of digital contact tracing technology in Germany and worldwide. The project started early in the pandemic, in March 2020, when mobile tracking apps were still at the development stage and public authorities were considering which technology to use (e.g., centralized vs. decentralized). After Germany launched the Corona-Warn-App in June 2020, we replaced the hypothetical scenarios by questions on using the app itself (see Supplementary Table B4 for details).

The second criterion was the development of the pandemic and changing infection numbers in Germany. The first sample was collected during the peak of the first wave of infections; the second, when infection rates were going down. Study Waves 3 and 4, focusing on the Corona-Warn-App, were likewise conducted at points with contrasting infection rates: in late summer 2020, when infections were low, and in November 2020, when they were rising steeply. We thus conducted two assessments when infections were peaking (Waves 1 and 4) and two when they were decreasing (Wave 2) or had been low for some time (Wave 3). For a visual representation, see Fig. 2.

**Figure 13:**
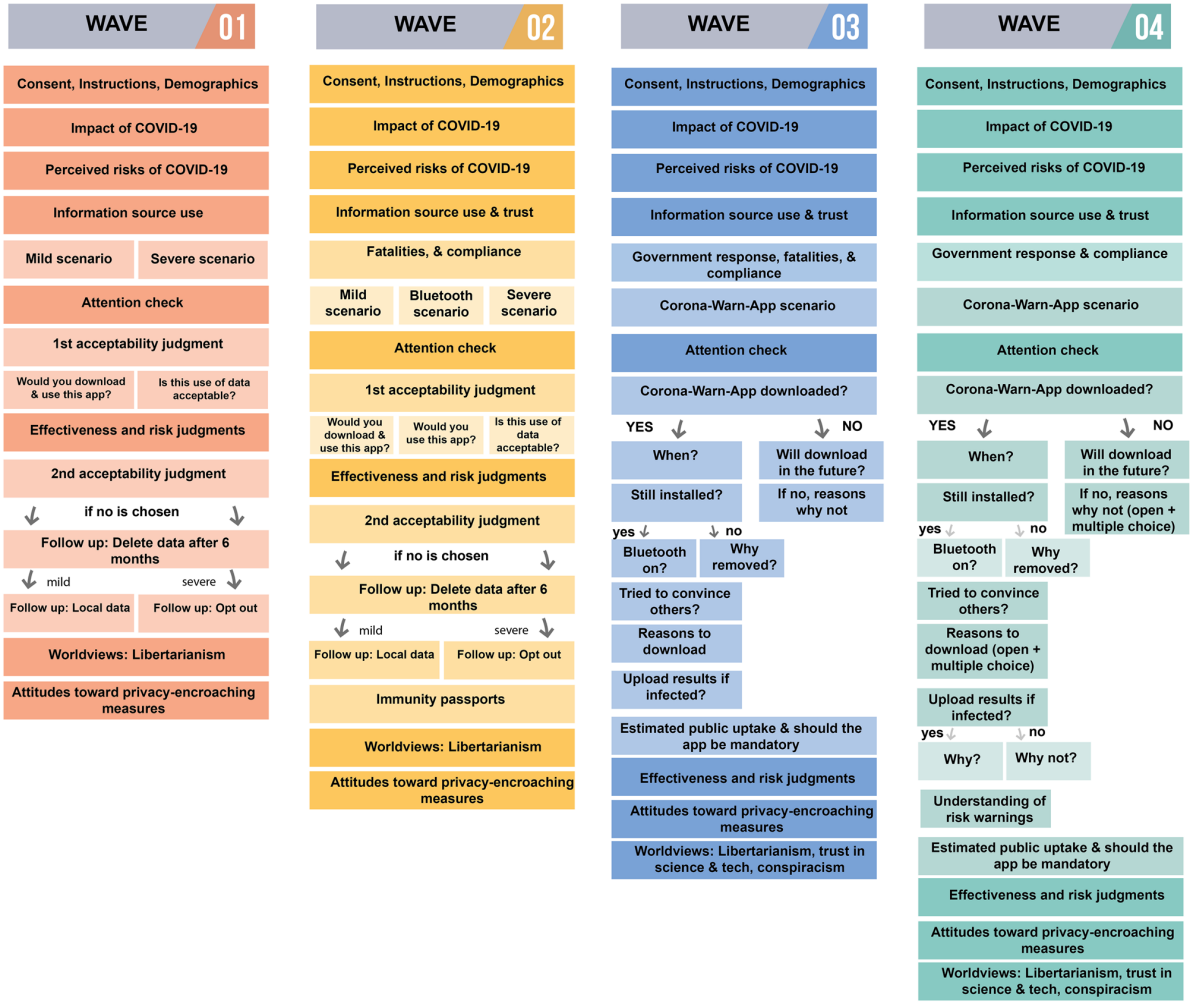
Design of Waves 1–4 of the survey. Each box represents a block of one or more questions. Blocks in deeper shades were common elements across all four waves. For the verbatim text of the scenarios (translated from the German), see Supplementary Table B4. Full questionnaires (in German) are available at https://osf.io/xvzph.

There were notable differences in the content of the four assessments (see Fig. 13 for a schematic representation), reflecting the ongoing developments in digital contact tracing technology. All surveys shared the same basic structure. Participants first completed an inventory of measures tapping the perceived impact and risks of COVID-19. They then saw one tracking scenario, followed by an inventory of measures tapping their attitudes toward the tracking technologies involved in the scenario seen. This inventory was the same across all scenarios, with one exception: Participants in Waves 1 and 2 were asked about a hypothetical app, whereas participants in Waves 3 and 4 were asked about the Corona-Warn-App itself. All participants also completed an attention check; those who failed to correctly identify the scenario they had seen from three alternatives were excluded from the analysis. The surveys concluded by assessing respondents’ political worldviews and their attitudes to hypothetical privacy-encroaching measures.

The details of these building blocks—perceived risks, scenario, attitudes toward the scenario presented, attention check, and worldview—differed somewhat between waves. Starting in Wave 2, we also included questions tapping participants’ assessment of the government’s response, estimation of fatalities, and compliance with social distancing rules. From Wave 3, we added further worldview items tapping, for example, attitudes to science and technology and belief in conspiracy narratives. The scenarios also differed between waves. In Waves 1 and 2, participants were randomly assigned to one of two (Wave 1) or three (Wave 2) hypothetical scenarios; in Waves 3 and 4, they saw a description of the Corona-Warn-App.

We now present individual blocks in more detail.

#### Impact of COVID-19

After providing consent and demographic information, respondents were asked about the impact of COVID-19 on themselves and people they know (see impact items in Supplementary Table B1).

#### Perceived risks of COVID-19

Respondents were then asked about how they perceived the risk of COVID-19 for themselves, other people, and the country as a whole (Risk items summarized in Supplementary Table B1).

#### Information source use and trust

They then were asked about which sources they used to inform themselves about the COVID-19 pandemic and how much they trusted these sources (see impact items in Supplementary Table B1).

#### Government response, fatalities, and compliance

Respondents then were asked to evaluate government response to the pandemic (Waves 3 and 4), to estimate national fatalities (Waves 2 and 3) and their own policy compliance (Waves 2–4) (see items in Supplementary Table B3).

Then they were randomly assigned to read a single tracking scenario description (in Waves 1 and 2) or proceeded to read the Corona-Warn-App description (in Waves 3 and 4).

#### Scenarios

The verbatim text of the scenarios (translated from the German) is provided in full in Supplementary Table B4. Here, we summarize the main differences.

#### Severe scenario

In this hypothetical scenario (Waves 1 and 2), anyone with a mobile phone would be tracked; there would be no possibility of opting out. The government could use the data to locate people who violate lockdown orders and fine or arrest them where necessary. Data would also be used to help shape the public health response, to contact people potentially exposed to COVID-19, and to issue individual quarantine orders.

#### Mild scenario

In this hypothetical scenario (Waves 1 and 2), only people who download a government app and agree to be tracked and contacted would be included. Data would only be used to contact those potentially exposed to COVID-19.

#### Bluetooth scenario

In this hypothetical scenario (Wave 2 only), respondents were informed about the Bluetooth-based contact tracing function proposed by Apple and Google. People potentially exposed to COVID-19 would be notified without the government knowing who they are. The use of this contact tracing capability would be completely voluntary. Those notified would not know the identity of the person who had tested positive.

#### Corona-Warn-App

In this scenario (Waves 3 and 4), respondents were informed about the Bluetooth-based app launched in Germany on 16 June 2020. Users who test positive for COVID-19 are asked whether they want to share their result for contact tracing. The app does not evaluate any geodata and does not transmit any location information. No personal data is sent or stored. The anonymized contact data is stored locally on the user’s smartphone.

#### Acceptability and uptake

In Waves 1 and 2, participants answered a series of questions assessing the acceptability of the scenario they had viewed, as well as their willingness to use the app described (for items, see Supplementary Table B5. Binary acceptability judgments (“Would you download and use the app?” for the mild and Bluetooth scenarios, and “Is this use of the tracking data acceptable?” for the severe scenario) were tapped twice: immediately after participants read the scenario and again after they had answered questions (standardized across waves) about the effectiveness and risks of the app presented. Participants who answered “No” after the second acceptability judgment were then asked follow-up questions probing whether their decision would change if the government (or Google and Apple in the Bluetooth scenario) were obliged to delete all data and stop tracking after 6 months. In the severe scenario, participants were also asked whether their decision would change if they had the option to opt out of data collection; in the mild scenario, they were asked if their decision would change if their data were only stored locally (see Supplementary Table B5 for all questions). In Waves 3 and 4, participants read the Corona-Warn-App scenario and were then asked whether they had downloaded the app or planned to download it in the future; they were also asked about their app usage and about the reasons for downloading/not downloading the app or uploading/not uploading their test results (multiple-choice format; multiple responses to this questions were allowed) (see Supplementary Table B5). In Wave 4, we asked participants to describe their reasons in their own words (open-response format) before presenting them with the same multiple-choice questions (see Supplementary Table B7).

#### Perceived effectiveness and risks of tracking technologies

Next block of questions probed respondents’ perceptions of the app’s effectiveness and potential risks (see Supplementary Table B6 for the full list of questions).

#### Worldviews

We collected information about participants’ worldviews, including attitudes toward the free market (based on^45,46^); higher mean responses reflected more conservative/libertarian worldviews. In Waves 3 and 4, we also surveyed respondents’ trust in science and endorsement of conspiracy beliefs. To measure conspiracy beliefs in Wave 3, we adapted a general conspiracy scale from^47^, selecting the five items with the highest item—total correlations and adding one additional item specifically tailored to the COVID-19 pandemic (“Selfish interests have conspired to convince the public that COVID-19 is a major threat,” designed based on the conspiracy beliefs inventory by Ref.^48^). In Wave 4, we created our own items based on COVID-19-related conspiracy narratives that were growing in popularity at the time. To counteract this exposure to conspiracy narratives, we included a debriefing flyer developed on the basis of^49^ recommendations at the end of the survey. For all worldview items, see Supplementary Table B8.

#### Attitudes toward privacy-encroaching measures

Finally, we asked respondents to rate the acceptability of six measures that could limit the spread of COVID-19. These hypothetical measures, which could potentially compromise people’s privacy, included giving the government access to medical records, tracking people’s locations using mobile phone data, and temporarily relaxing data protection regulations (for a full list, see Supplementary Table B9).

### Data analysis and reporting

We used logistic regression analyses to identify predictors of Corona-Warn-App downloads in Waves 3 and 4 of the survey. All combined measures used in the regressions analyses are summarized in Supplementary Table B10. For regression analyses, gender coefficient was assessed as a binary variable, and “Other” category was excluded from regression analyses due to its small sample size (see Table 1). To analyze the open-response question on why people did or did not download the app, we counted the frequencies with which terms occurred across different respondents’ responses and the frequency with which the terms co-occurred within the same respondent’s response. Based on these frequencies we built co-occurrence networks of unigrams (i.e., individual words) using a simple feature extraction method from the Python package scikit-learn (version 0.24.1) to collect unigram frequencies^50^; we used the graph-tool library (version 2.37) to build networks of unigrams according to their co-occurrences within a response^51^. In this article, we report selected results relevant for understanding public attitudes toward privacy and tracking technologies during the pandemic. Full descriptive results for all four waves of the survey are available online: https://ai_society.mpib.dev/tracking-app.

## Supplementary Information

Supplementary Information.

## Data Availability

Anonymized data and code, as well as the German version of the study questionnaires are available at (https://osf.io/xvzph).
