# Supplementary material for: Psychological factors shaping public responses to COVID-19 digital contact tracing technologies in Germany
Source: Sci Rep. 2021 Sep 21;11:18716. doi: 10.1038/s41598-021-98249-5 (PMC8455538; doi:10.1038/s41598-021-98249-5)
Supplement: Supplementary file 1 — Supplementary Information. [file 41598_2021_98249_MOESM1_ESM.pdf]

Supplementary Information for: Psychological Factors Shaping Public Responses to COVID-19 Digital Contact Tracing Technologies in Germany

Anastasia Kozyreva[1*], Philipp Lorenz-Spreen[1], Stephan Lewandowsky[2,3], Paul M. Garrett[4], Stefan M. Herzog[1], Thorsten Pachur[1], and Ralph Hertwig[1]

[1]Center for Adaptive Rationality, Max Planck Institute for Human Development, Germany

[2]School of Psychological Science, University of Bristol, United Kingdom

[3]School of Psychological Sciences, University of Western Australia

[4]Melbourne School of Psychological Sciences, University of Melbourne, Australia

[*]Corresponding author, E-mail: kozyreva@mpib-berlin.mpg.de

Supplementary Information for: Psychological Factors Shaping Public Responses to COVID-19 Digital Contact Tracing Technologies in Germany

Appendix A: Supplementary Information: Figures and Tables (pp. 3–20)

- Figures A1–A11

- Tables A1–A10

Appendix B: Supplementary Information: Items and Covariates (pp. 21–28)

- Tables B1–B10

Appendix A

Supplementary Information: Figures and Tables

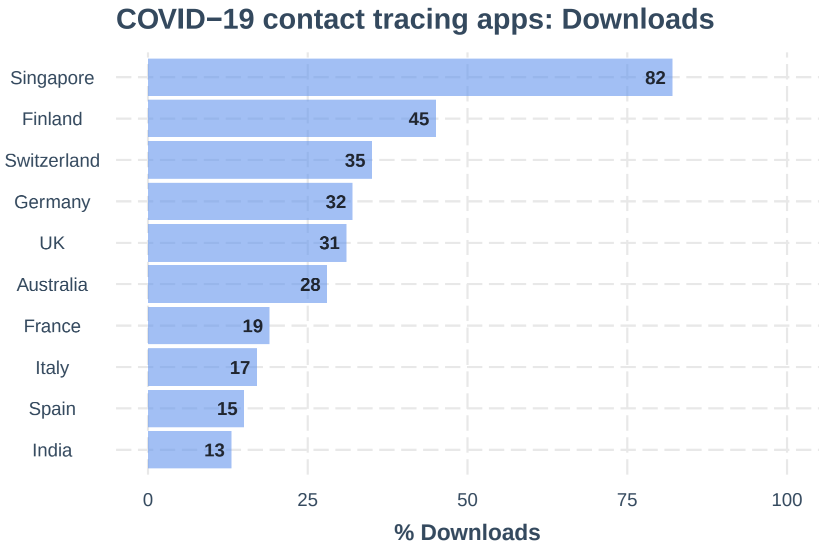

Figure A1: Adoption of COVID-19 contact tracing apps in selected countries, as indicated by the percentage of the population that has downloaded the app. See Table A1 for detailed information. Latest update: April 7, 2021.

Table A1

Adoption of COVID-19 Contact Tracing Apps in Selected Countries: Further Information on Apps and Download Figures

Country	Name	Developer/Deployer	Technology	Release date	Downloads (N)	Downloads (%)	Numbers updated on
Singapore	TraceTogether	GovTech Agency	Bluetooth, BlueTrace	20.03.2020	4,700,000	82%	07.04.2021*
Finland	Koronavilkku	Finnish Institute of Health and Welfare	Bluetooth, Google/Apple	31.08.2020	2,500,000	45%	05.11.2020**
Switzerland	SwissCovid	Swiss National Covid-19 Science Task Force	Bluetooth, Google/Apple	23.07.2020	3,059,000	35%	06.04.2021
Germany	Corona-Warn-App	Deutsche Telekom, SAP/Robert Koch Institute	Bluetooth, Google/Apple	16.06.2020	26,700,000	32%	01.04.2021
United Kingdom	NHS COVID-19 App	NHS	Bluetooth, Google/Apple	24.09.2020	20,900,000	31%	23.12.2020**
Australia	COVIDSafe	Australian government	Bluetooth	26.04.2020	7,000,000	28%	07.04.2020*
France	TousAntiCovid	Inria	Bluetooth	22.10.2020	13,000,000	19%	01.03.2021**
Italy	Immuni	Bending Spoons	Bluetooth, Google/Apple	01.06.2020	10.400.709	17%	01.04.2021
Spain	Radar COVID	Ministry of Economic Affairs and Digital Transformation	Bluetooth, Google/Apple	August 2020	7,200,000	15%	28.03.2021
India	Aarogya Setu	Indian national government	Bluetooth, Location	02.04.2020	173,700,000	13%	07.04.2020

* The app's website provides only approximate numbers of downloads and no information about when the numbers were updated. ** The app's website provides no information about downloads; the reported numbers are taken from press coverage or statista.com.

Table A2

Cochran–Armitage Trend Test for Figure 4

Measure	n	χ^2	p value	df
Data on infections & immunity status	4302	24.208	<.001	1
Access to medical records	4302	239.253	<.001	1
Location tracking data	4302	84.693	<.001	1
Notifications of leaving quarantine	4302	16.747	<.001	1
Suspension of data protection	4302	6.674	.01	1
Data on contacts & interactions	4302	5.883	.02	1

Table A3

1-Sample Proportions Test with Continuity Correction (`prop.test` in R) for Figure 4

Measure	Wave	N accept	% accept	Total N	95% CI (low)	95% CI (high)
Data on contacts & interactions	Wave 1	387	49	788	0.46	0.53
Suspension of data protection	Wave 1	409	52	788	0.48	0.55
Data on infections & immunity status	Wave 1	541	69	788	0.65	0.72
Location tracking data	Wave 1	470	60	788	0.56	0.63
Access to medical records	Wave 1	533	68	788	0.64	0.71
Notifications of leaving quarantine	Wave 1	465	59	788	0.55	0.62
Data on contacts & interactions	Wave 2	498	45	1102	0.42	0.48
Suspension of data protection	Wave 2	525	48	1102	0.45	0.51
Data on infections & immunity status	Wave 2	719	65	1102	0.62	0.68
Location tracking data	Wave 2	605	55	1102	0.52	0.58
Access to medical records	Wave 2	649	59	1102	0.56	0.62
Notifications of leaving quarantine	Wave 2	549	50	1102	0.47	0.53
Data on contacts & interactions	Wave 3	453	37	1230	0.34	0.40
Suspension of data protection	Wave 3	548	45	1230	0.42	0.47
Data on infections & immunity status	Wave 3	669	54	1230	0.52	0.57
Location tracking data	Wave 3	576	47	1230	0.44	0.50
Access to medical records	Wave 3	590	48	1230	0.45	0.51
Notifications of leaving quarantine	Wave 3	637	52	1230	0.49	0.55
Data on contacts & interactions	Wave 4	536	45	1182	0.42	0.48
Suspension of data protection	Wave 4	547	46	1182	0.43	0.49
Data on infections & immunity status	Wave 4	714	60	1182	0.58	0.63
Location tracking data	Wave 4	481	41	1182	0.38	0.44
Access to medical records	Wave 4	413	35	1182	0.32	0.38
Notifications of leaving quarantine	Wave 4	565	48	1182	0.45	0.51

Table A4

1-Sample Proportions Test With Continuity Correction (prop.test in R) for Figure 5

Scenario type	Wave	Item	N accept	% accept	Total N	95% CI (low)	95% CI (high)
Severe	Wave 1	Acceptability of the scenario	238	56	425	0.51	0.61
Severe	Wave 1	With follow up: delete data	292	69	425	0.64	0.73
Severe	Wave 1	With follow up: opt out	334	79	425	0.74	0.82
Severe	Wave 2	Acceptability of the scenario	223	55	407	0.50	0.60
Severe	Wave 2	With follow up: delete data	280	69	407	0.64	0.73
Severe	Wave 2	With follow up: opt out	327	80	407	0.76	0.84
Mild	Wave 1	Acceptability of the scenario	245	61	404	0.56	0.65
Mild	Wave 1	With follow up: delete data	287	71	404	0.66	0.75
Mild	Wave 1	With follow up: local data	300	74	404	0.70	0.78
Mild	Wave 2	Acceptability of the scenario	232	64	362	0.59	0.69
Mild	Wave 2	With follow up: delete data	259	72	362	0.67	0.76
Mild	Wave 2	With follow up: local data	286	79	362	0.74	0.83
Bluetooth	Wave 2	Acceptability of the scenario	200	59	340	0.53	0.64
Bluetooth	Wave 2	With follow up: delete data	228	67	340	0.62	0.72
Corona-Warn-App	Wave 3	Downloads	446	36	1231	0.34	0.39
Corona-Warn-App	Wave 3	With follow up: future downloads	634	52	1231	0.49	0.54
Corona-Warn-App	Wave 4	Downloads	483	41	1188	0.38	0.44
Corona-Warn-App	Wave 4	With follow up: future downloads	657	55	1188	0.52	0.58

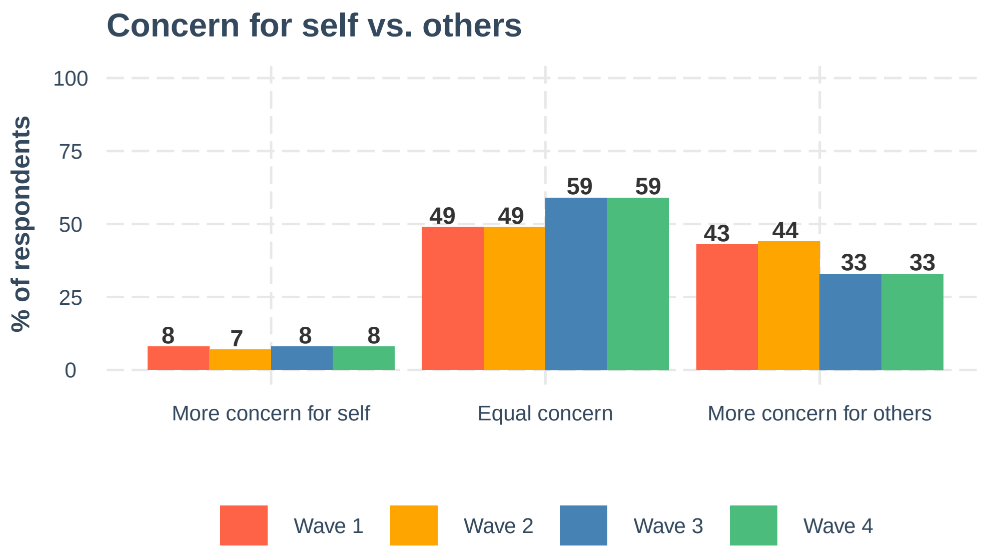

Figure A2: Concern for self and others: COVID-19 risk perceptions within respondents. Responses are grouped into three categories: (1) respondents who rated concern for themselves higher than concern for others, (2) respondents who gave the same rating to both, and (3) respondents who rated concern for others higher than concern for themselves. Questions: (1) Concern self: How concerned are you that you might become infected with COVID-19? (2) Concern others: How concerned are you that somebody you know might become infected with COVID-19?"

Table A5
Corona-Warn-App Usage

	Wave 3	Wave 4
**Have you downloaded the Corona-Warn-App?**		
Yes	36.2	40.7
No	63.8	59.3
**Is the Corona-Warn-App still installed on your phone?**		
Yes	91.6	93.2
No	8.4	6.8
**Do you generally have Bluetooth switched on so the Corona-Warn-App can operate effectively?**		
Yes	76.9	74.4
No	3.3	4.9
Only when I leave the house	18.0	18.4
I don't know	1.8	2.2
**Have you made any attempts to convince your friends and/or family to download the Corona-Warn-App?**		
Yes	73.1	67.3
No	26.9	32.7
**Will you download the Corona-Warn-App in the future?**		
Yes	23.9	24.7
No	76.1	75.3
**Do you think that the government should make the Corona-Warn-App mandatory?**		
Yes	28.8	30.1
No	71.2	69.9

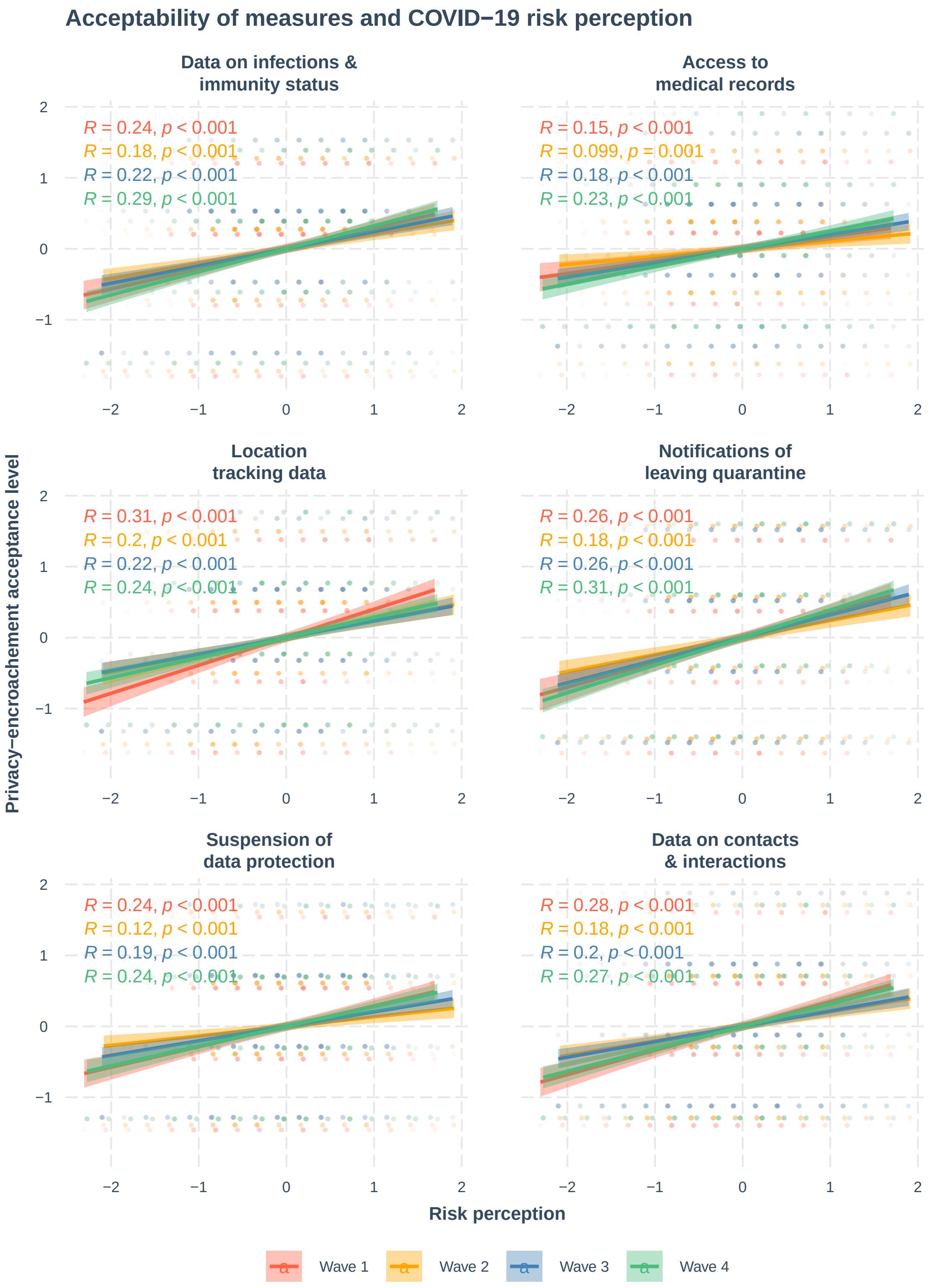

Figure A3: Correlations between acceptability of various privacy-encroaching measures and COVID-19 risk perceptions within respondents across all four waves of the survey. The risk perception variable is a combined score for the four items in Figure 3. All variables are center-scaled. Individual responses are jittered. Lines represent simple linear regression slopes and their 95% confidence band.

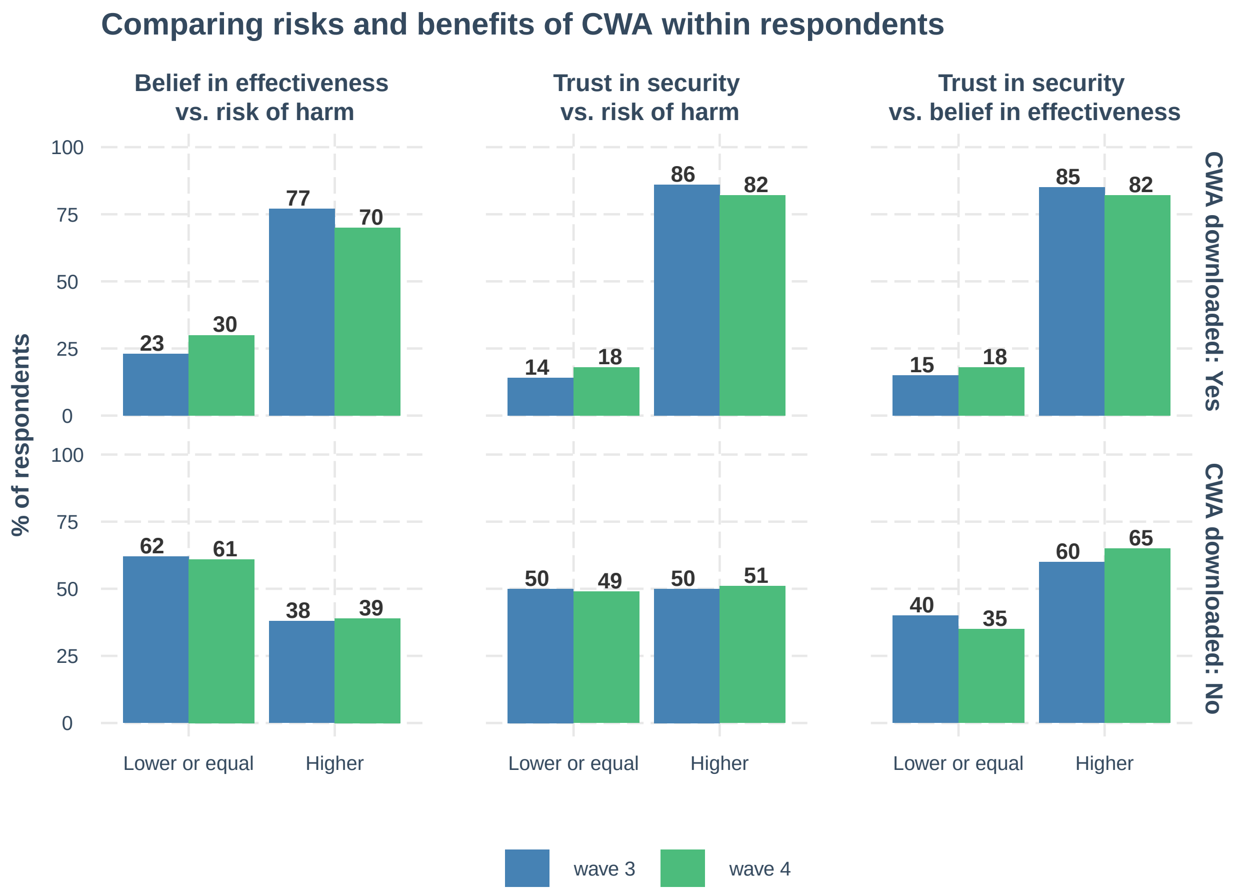

Figure A4: Within-participants comparisons of perceived effectiveness and risks of the Corona-Warn-App. The variables "Trust in security" and "Belief in effectiveness" represent combined and averaged measures from variables presented in Figure 6. The variable "Trust in security" combines four variables: "Trust: Data for Pandemic Only," "Trust: Privacy Protection," "Only Necessary Data Collected" "Trust: Security from 3rd Party." The variable "Belief in effectiveness" combines three variables: "Reduce Likelihood to Contract," "Reduce Spread Future," "Return to Activity Future." "Risk of harm" is a single variable. For items, see Appendix Tables B6. The figure complements Figure 7.A and shows comparisons on the individual level.

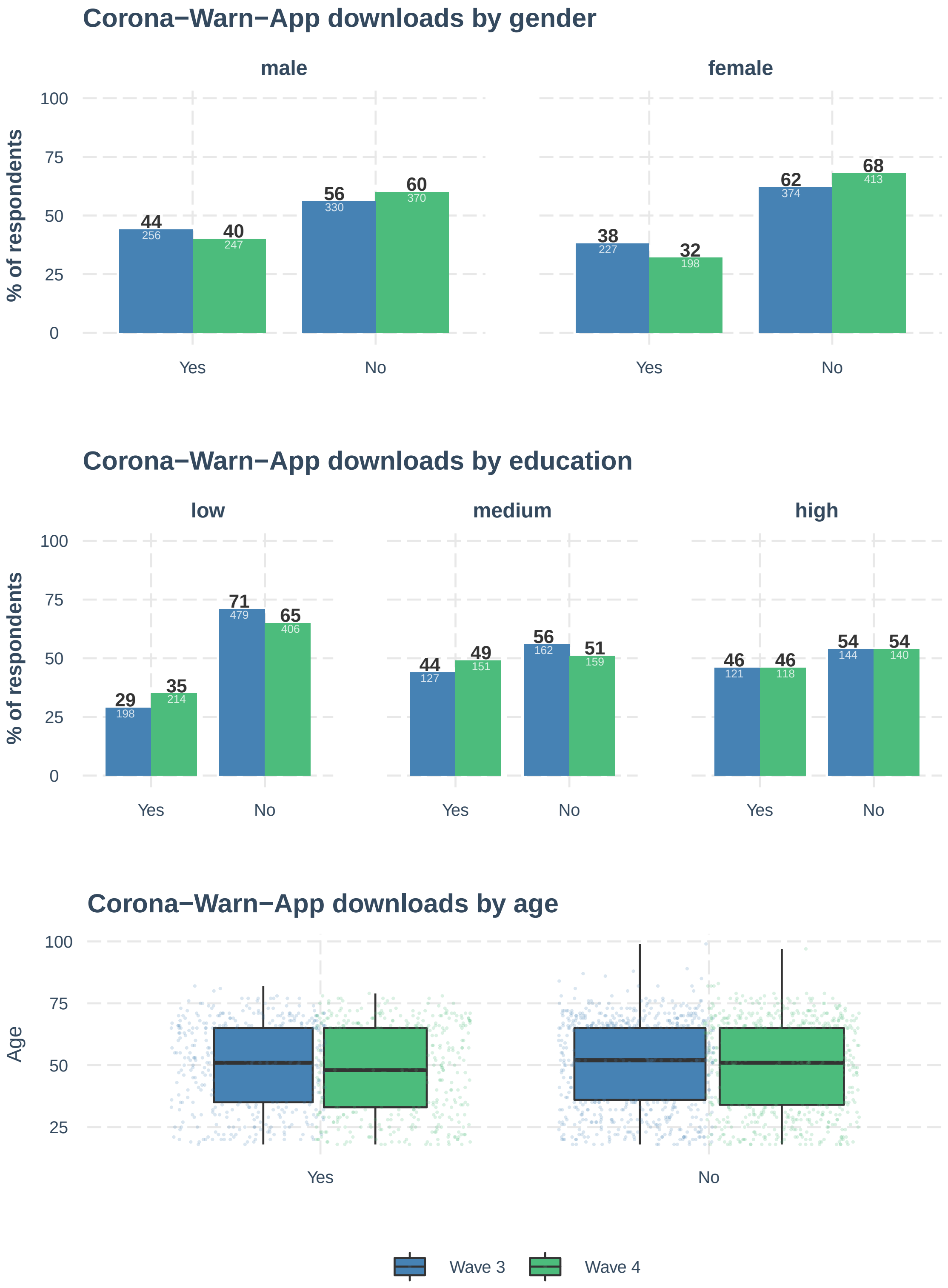

Figure A5: Reported Corona-Warn-App downloads by demographic characteristics. For education, "low" comprises the categories "*Realschule*," "*Hauptschule*," and "None" as the highest level of education; "medium" indicates "*Abitur*;" and "high" indicates "University". For gender, the "Other" category was excluded from this analysis due to its small sample size (see Table 1). Barplots: Percentages shown in black; respondent numbers shown in white. Boxplots: Boxes show the interquartile range (IQR) of the age distribution (values between the 25th and 75th percentiles); the black lines inside the boxes indicate the median value. Lower and upper whiskers extend from the hinge to the smallest and largest values within $1.5 \times IQR$. Individual responses are jittered both horizontally and vertically.

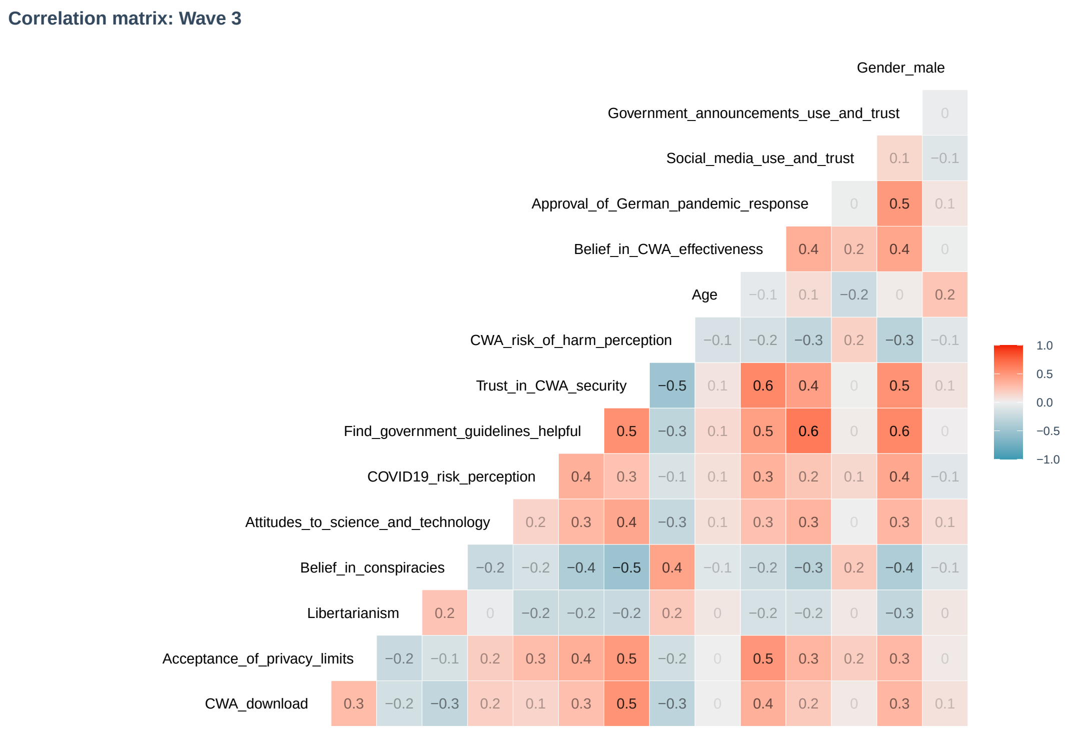

Figure A6: Pearson correlation matrix for variables in Figure 10, Wave 3. Positive correlations are displayed in red, negative correlations in blue, and small correlations in gray. Color intensity is proportional to the correlation coefficients.

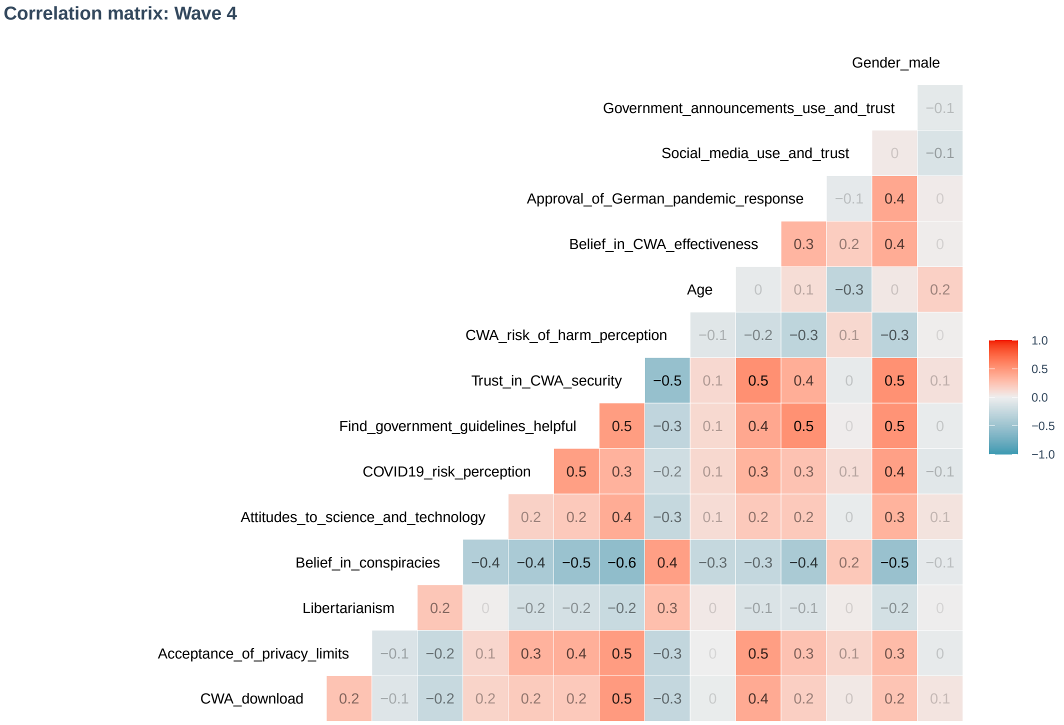

Figure A7: Pearson correlation matrix for variables in Figure 10, Wave 4. Positive correlations are displayed in red, negative correlations in blue, and small correlations in gray. Color intensity is proportional to the correlation coefficients.

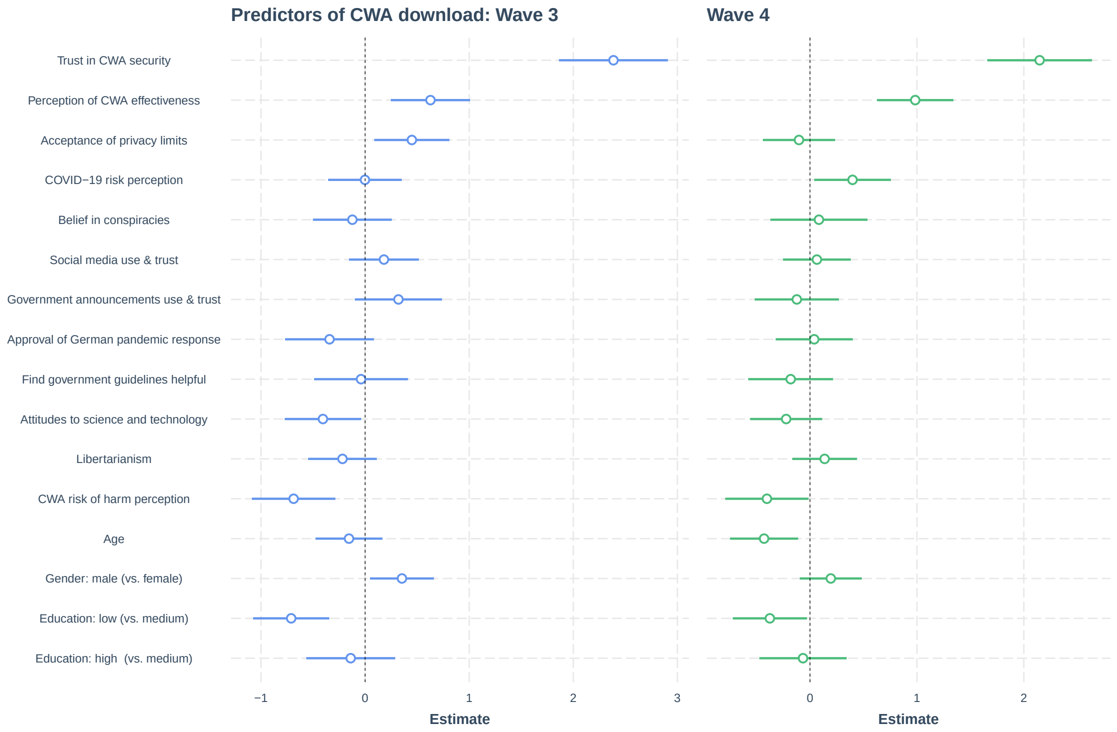

Figure A8: Logistic regression models predicting Corona-Warn-App download in Waves 3 and 4 (an alternative representation of Figure 10). Dependent variable: downloaded the app (yes/no). Coefficients: measures from the survey (e.g., combined score for conspiracy beliefs; see Appendix Table B10). Horizontal point ranges indicate point estimates and 95% confidence intervals for each predictor. Education was dummy coded with the reference level "medium," yielding two coefficients: low (vs. medium) and high (vs. medium) education. Following Gelman (2008), we standardized all continuous variables by two standard deviations (SD) and mean centered the binary gender variable. This way a 2-SD change in a continuous predictor variable is approximately equivalent to a change of category in a roughly balanced binary predictor variable (e.g., gender). In a logistic regression model, a slope reflects the relative change in log odds (while keeping all other predictors at their average values). Appendix Table A6 summarizes the regression results for these two models. Appendix Figures A6 and A7 display Pearson correlations for all variables in the regression model.

Table A6

Regression Results for Figure 10 and Appendix Figure A8: Predictors of Corona-Warn-App (CWA) Downloads. Numbers represent point estimates and 95% confidence intervals for each predictor.

	Dependent variable:	
	CWA downloads	
	Wave 3	Wave 4
	(1)	(2)
Trust in CWA security	2.386	2.148
	(1.862,2.910)	(1.657,2.638)
Perception of CWA effectiveness	0.628	0.984
	(0.247,1.009)	(0.627,1.342)
Acceptance of privacy limits	0.449	−0.104
	(0.088,0.810)	(-0.441,0.234)
Approval of German pandemic response	−0.342	0.039
	(-0.769,0.085)	(-0.322,0.400)
Find government guidelenes helpful	−0.039	−0.181
	(-0.490,0.412)	(-0.578,0.216)
Social media use and trust	0.181	0.064
	(-0.155,0.517)	(-0.253,0.382)
Government announcements use and trust	0.320	−0.124
	(-0.099,0.739)	(-0.517,0.270)
Belief in conspiracies	−0.122	0.083
	(-0.501,0.257)	(-0.371,0.538)
Libertarianism	−0.217	0.137
	(-0.547,0.112)	(-0.166,0.439)
COVID-19 risk perception	−0.001	0.398
	(-0.355,0.352)	(0.039,0.756)
Attitudes to science and technology	−0.405	−0.223
	(-0.773,-0.038)	(-0.560,0.114)
Gender: male (vs. female)	0.354	0.194
	(0.047,0.661)	(-0.096,0.485)
Age	−0.155	−0.430
	(-0.477,0.168)	(-0.748,-0.111)
CWA risk of harm perception	−0.687	−0.403
	(-1.088,-0.285)	(-0.793,-0.013)
Education: low (vs. medium)	−0.710	−0.376
	(-1.076,-0.344)	(-0.723,-0.029)
Education: high (vs. medium)	−0.138	−0.066
	(-0.565,0.289)	(-0.474,0.343)
Constant	−0.575	−0.422
	(-0.876,-0.275)	(-0.707,-0.136)
Observations	1,183	1,140

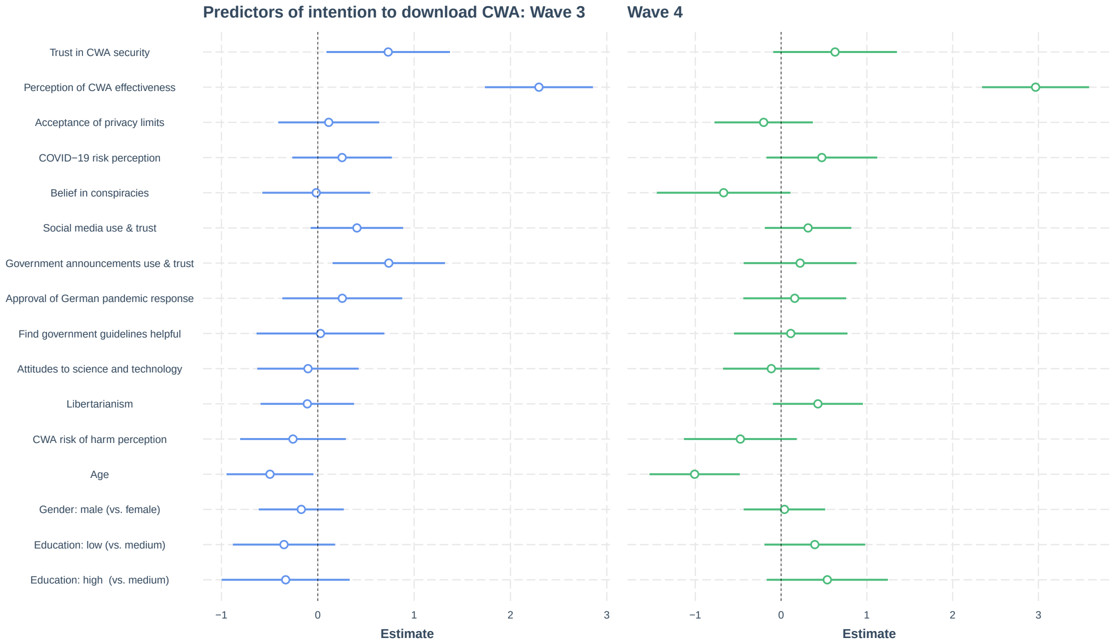

Figure A9: Logistic regression models predicting intention to download the Corona-Warn-App in Waves 3 and 4 (an alternative representation of Figure 11). Dependent variable: downloaded the app (yes/no). Coefficients: measures from the survey (e.g., combined score for CWA in app security; combined score for conspiracy beliefs; see Appendix Table B10). Horizontal point ranges show point estimates and 95% confidence intervals for each predictor. Education was dummy coded with the reference level "medium," yielding two coefficients: low (vs. medium) and high (vs. medium) education. Following Gelman (2008), we standardized all continuous variables by two standard deviations (SD) and mean centered the binary gender variable. This way a 2-SD change in a continuous predictor variable is approximately equivalent to a change of category in a roughly balanced binary predictor variable (e.g., gender). In a logistic regression model, a slope reflects the relative change in log odds (while keeping all other predictors at their average values). Appendix Table A6 summarizes the regression results for these two models. Appendix Figures A6 and A7 display Pearson correlations for all variables in the regression model.

Table A7

Regression Results for Figure 11 and Appendix Figure A9: Predictors of Intention to Download the Corona-Warn-App (CWA). Numbers represent point estimates and 95% confidence intervals for each predictor.

	Dependent variable:	
	CWA downloads	
	Wave 3	Wave 4
	(1)	(2)
Trust in CWA security	0.731	0.629
	(0.090,1.372)	(-0.092,1.350)
Perception of CWA effectiveness	2.296	2.967
	(1.735,2.856)	(2.343,3.591)
Acceptance of privacy limits	0.114	−0.203
	(-0.410,0.637)	(-0.777,0.371)
Approval of German pandemic response	0.254	0.159
	(-0.368,0.876)	(-0.441,0.758)
Find government guidelines helpful	0.028	0.113
	(-0.636,0.691)	(-0.549,0.774)
Social media use and trust	0.406	0.315
	(-0.074,0.886)	(-0.190,0.819)
Government announcements use and trust	0.737	0.222
	(0.154,1.320)	(-0.436,0.880)
Belief in conspiracies	−0.016	−0.670
	(-0.576,0.544)	(-1.449,0.109)
Libertarianism	−0.109	0.429
	(-0.594,0.376)	(-0.095,0.954)
COVID-19 risk perception	0.252	0.475
	(-0.265,0.769)	(-0.171,1.120)
Attitudes to science and technology	−0.102	−0.114
	(-0.628,0.424)	(-0.677,0.449)
Gender: male (vs. female)	−0.171	0.039
	(-0.613,0.270)	(-0.436,0.515)
Age	−0.496	−1.008
	(-0.947,-0.045)	(-1.535,-0.482)
CWA risk of harm perception	−0.258	−0.475
	(-0.806,0.291)	(-1.132,0.183)
Education: low (vs. medium)	−0.351	0.393
	(-0.881,0.180)	(-0.195,0.980)
Education: high (vs. medium)	−0.333	0.539
	(-0.998,0.332)	(-0.168,1.245)
Constant	−1.526	−2.219
	(-2.001,-1.050)	(-2.782,-1.656)
Observations	758	684

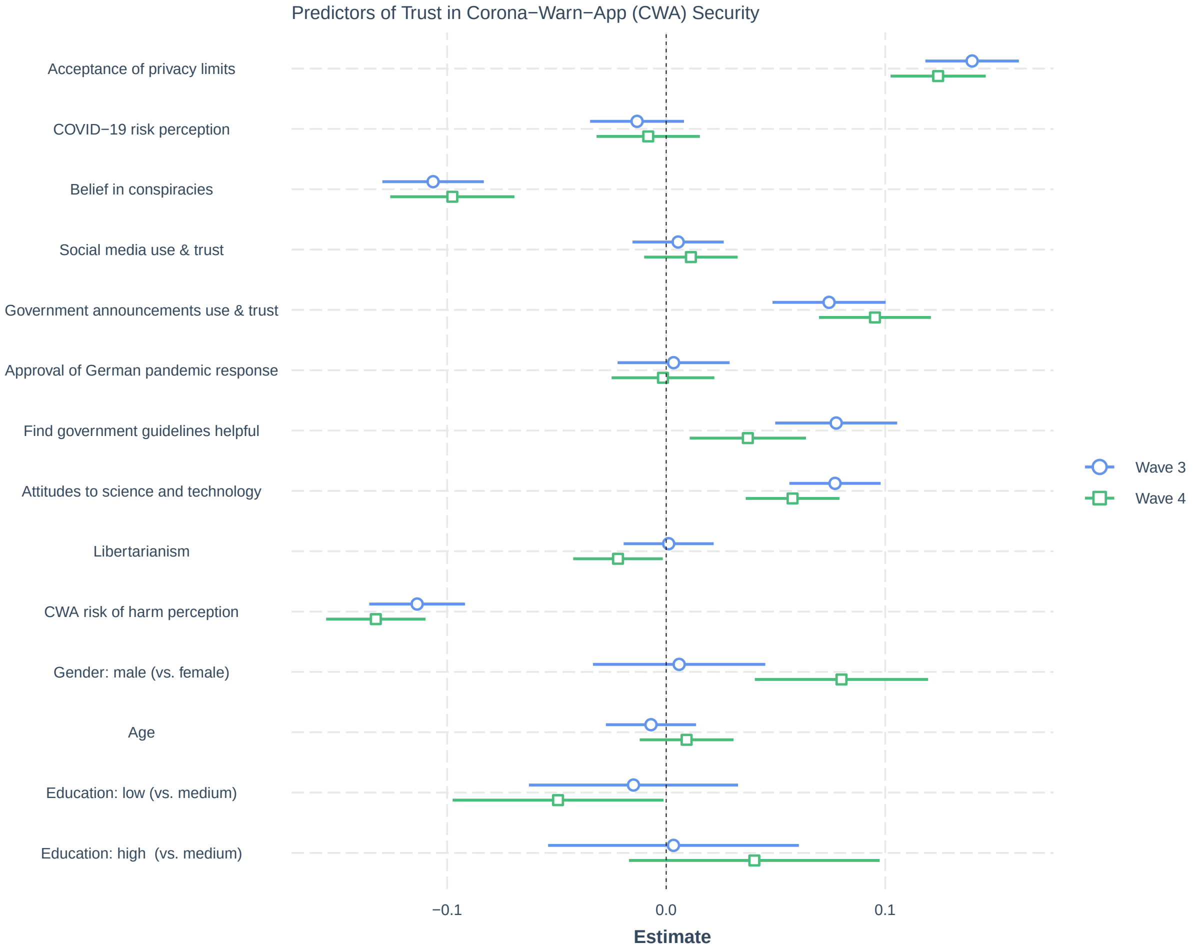

Figure A10: Linear regression models predicting trust in Corona-Warn-App security in Waves 3 and 4. Dependent variable: Trust in CWA security. Coefficients: various measures from the survey (e.g., combined score for conspiracy beliefs; see Appendix Table B10). Horizontal point ranges show point estimates and 95% confidence intervals for each predictor. Education was dummy coded with the reference level "medium" yielding two coefficients: low (vs. medium) and high (vs. medium) education. Following Gelman (2008), we standardized all continuous variables and the dependent variable by two standard deviations (SD) and mean centered binary variables. This way a 2-SD change in a continuous predictor variable is approximately equivalent to a change of category in a roughly balanced binary predictor variable (e.g., gender). Furthermore, because we also standardized the dependent variable by $2SD$, a slope of, say, $+0.1$ can be interpreted as follows: If the predictor is increased by, for instance, $1SD$ of its distribution, the dependent variable increases by $0.1SD$ of its distribution (while keeping all other predictors at their average values). Appendix Table A8 summarizes the regression results for these two models.

Table A8

Regression Results for Appendix Figure A10: Predictors of Trust in Corona-Warn-App Security in Waves 3 and 4. Numbers represent point estimates and 95% confidence intervals for each predictor.

	Dependent variable:	
	Trust in Corona-Warn-App security	
	Wave 3	Wave 4
	(1)	(2)
Acceptance of privacy limits	0.279	0.248
	(0.237,0.322)	(0.205,0.292)
Approval of German pandemic response	0.007	−0.003
	(-0.044,0.058)	(-0.050,0.044)
Find government guidelines helpful	0.155	0.075
	(0.100,0.211)	(0.021,0.128)
Social media use and trust	0.011	0.023
	(-0.031,0.053)	(-0.020,0.065)
Government announcements use and trust	0.149	0.191
	(0.097,0.200)	(0.140,0.242)
Belief in conspiracies	−0.213	−0.195
	(-0.259,-0.167)	(-0.252,-0.139)
Libertarianism	0.002	−0.044
	(-0.039,0.043)	(-0.085,-0.003)
COVID-19 risk perception	−0.027	−0.016
	(-0.069,0.016)	(-0.064,0.031)
Attitudes to science and tech	0.154	0.115
	(0.113,0.196)	(0.073,0.158)
CWA risk of harm perception	−0.227	−0.265
	(-0.271,-0.184)	(-0.310,-0.220)
Gender: male (vs. female)	0.006	0.080
	(-0.033,0.045)	(0.041,0.120)
Age	−0.014	0.019
	(-0.055,0.027)	(-0.024,0.061)
Education: low (vs. medium)	−0.015	−0.049
	(-0.063,0.033)	(-0.097,-0.001)
Education: high (vs. medium)	0.003	0.040
	(-0.054,0.061)	(-0.017,0.097)
Constant	0.007	0.017
	(-0.032,0.047)	(-0.022,0.056)
Observations	1,183	1,140
R^2	0.575	0.572
Adjusted R^2	0.570	0.566

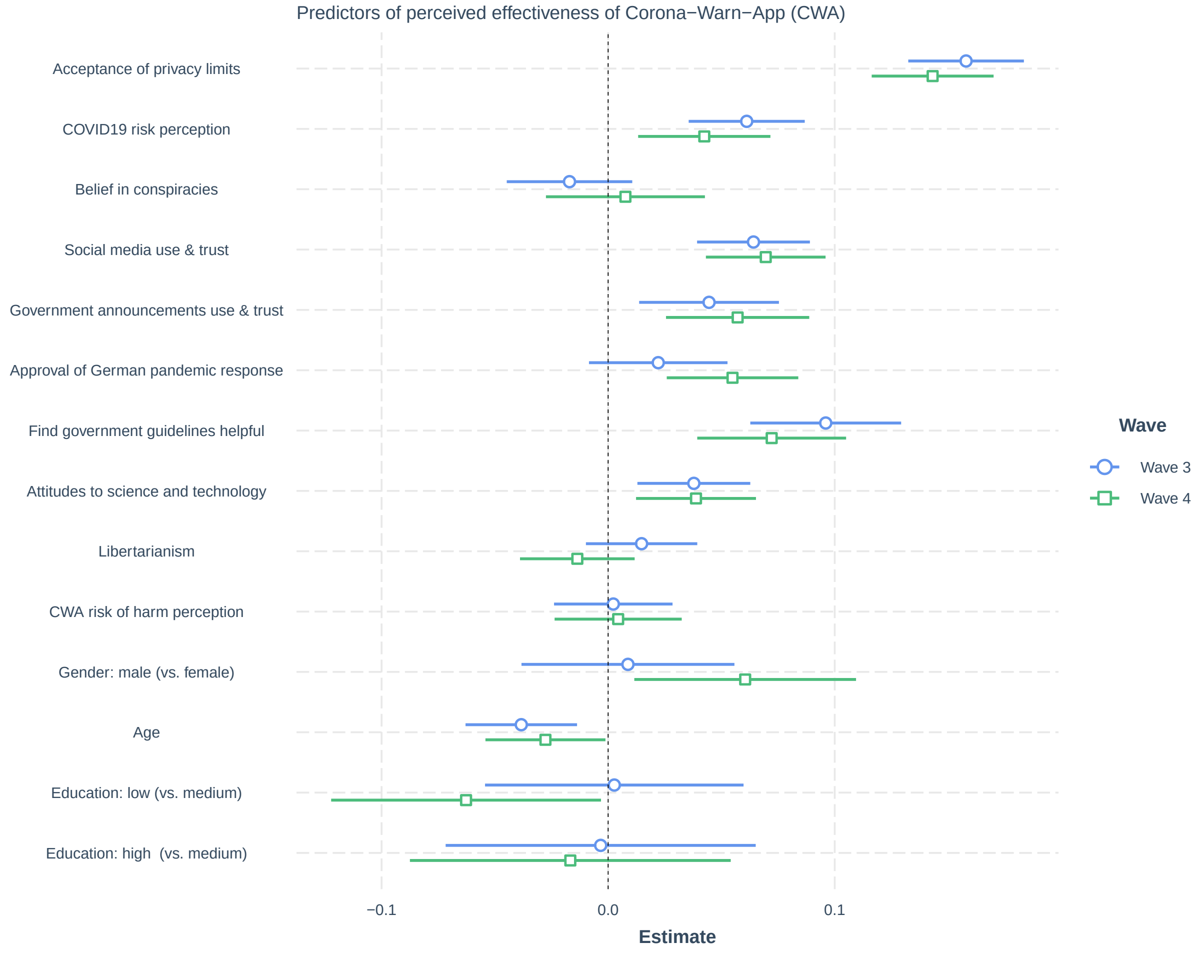

Figure A11: Linear regression models predicting perceived effectiveness of the Corona-Warn-App in Waves 3 and 4. Dependent variable: Perceived effectiveness of the Corona-Warn-App. Coefficients: various measures from the survey (e.g., combined score for trust in CWA security; combined score for conspiracy beliefs; see Appendix Table B10). Education was dummy coded with the reference level "medium," yielding two coefficients: low (vs. medium) and high (vs. medium) education. Following Gelman (2008), we standardized all continuous variables and the dependent variable by two standard deviations (SD) and mean centered binary variables. This way a 2-SD change in a continuous predictor variable is approximately equivalent to a change of category in a roughly balanced binary predictor variable (e.g., gender). Furthermore, because we also standardized the dependent variable by $2SD$, a slope of, say, +0.1 can be interpreted as follows: If the predictor is increased by, for instance, $1SD$ of its distribution, the dependent variable increases by $0.1SD$ of its distribution (while keeping all other predictors at their average values). Appendix Table A9 summarizes the regression results for these models.

Table A9

Regression Results for Appendix Figure A11: Predictors of Perceived Effectiveness of Corona-Warn-App in Waves 3 and 4. Numbers represent point estimates and 95% confidence intervals for each predictor.

	Dependent variable:	
	Perceived effectiveness of the Corona-Warn-App	
	Wave 3	Wave 4
	(1)	(2)
Acceptance of privacy limits	0.316	0.286
	(0.265,0.367)	(0.233,0.340)
Approval of German pandemic response	0.044	0.110
	(-0.017,0.105)	(0.052,0.168)
Find governement guidelenes helpful	0.192	0.144
	(0.126,0.259)	(0.079,0.210)
Social media use and trust	0.128	0.139
	(0.079,0.178)	(0.086,0.192)
Governement announcements use and trust	0.089	0.114
	(0.027,0.151)	(0.051,0.177)
Belief in conspiracies	−0.034	0.015
	(-0.089,0.021)	(-0.055,0.085)
Libertarianism	0.030	−0.027
	(-0.020,0.079)	(-0.078,0.023)
Covid-19 risk perception	0.122	0.085
	(0.071,0.173)	(0.027,0.143)
Attitudes to science and tech	0.076	0.078
	(0.026,0.125)	(0.025,0.130)
CWA risk of harm perception	0.005	0.009
	(-0.048,0.057)	(-0.047,0.065)
Gender: male (vs. female)	0.009	0.060
	(-0.038,0.056)	(0.012,0.109)
Age	−0.077	−0.055
	(-0.126,-0.027)	(-0.108,-0.002)
Education: low (vs. medium)	0.003	−0.063
	(-0.054,0.060)	(-0.122,-0.003)
Education: high (vs. medium)	−0.003	−0.017
	(-0.072,0.065)	(-0.087,0.054)
Constant	−0.001	0.037
	(-0.048,0.046)	(-0.012,0.085)
Observations	1,183	1,140
R^2	0.394	0.344
Adjusted R^2	0.386	0.336

Table A10
Demographic Information: Education and Region

	Wave 1	Wave 2	Wave 3	Wave 4
**Sample size**				
N	829	1,109	1,231	1,188
**Education (highest level) (%)**				
University	25.8	23.2	21.5	21.7
Abitur (academic track)	26.8	27.8	23.5	26.1
Realschule (intermediate track)	33.3	35.3	36.6	36.1
Hauptschule (vocational track)	13.5	13.1	17.6	15.5
None	0.6	0.7	0.7	0.6
**Region (%)**				
Bremen, Hamburg, Lower Saxony, Schleswig-Holstein	16.2	16.3	16.5	16.6
North Rhine-Westphalia	22.7	22.2	21.3	23.1
Hesse, Rhineland-Palatinate, Saarland	13.9	15.6	14.5	13.3
Baden-Württemberg	11.2	9.8	12.0	10.4
Bavaria	14.8	14.6	14.6	14.8
Berlin, Brandenburg, Mecklenburg-Vorpommern, Saxony-Anhalt	13.8	14.2	13.4	13.8
Saxony, Thuringia	7.5	7.3	7.7	8.0

Appendix B

Supplementary Information: Items and Covariates

Table B1

Items Assessing Impact of COVID-19, and Information Use and Trust

Question	Answer options
**Impact of COVID-19**	
Have you tested positive for COVID-19?	Yes/No
Do you know anyone who has tested positive for COVID-19?	Yes/No
How many days, if any, did you voluntarily go into quarantine?	Open numeric response on slider scale
Have you temporarily or permanently lost your job as a consequence of the COVID-19 pandemic?	Yes/No
**Information Use and Trust**	
How often do you rely on the following media to stay informed about developments surrounding the COVID-19 pandemic? Please select all media you use: Newspapers (also online); Social media; Friends and family; Radio; Television; Government announcements (including their websites)	5-Point Likert Scale (1 = Never, 5 = Always)
How would you rate the following sources? Do you think the information about the COVID-19 pandemic is accurate and trustworthy? Newspapers (also online); Social media; Friends and family; Radio; Television; Government announcements (including their websites)	5-Point Likert Scale (1 = Not at All, 5 = Completely)

Table B2

Items Assessing Perceived Risks of COVID-19 on a 5-Point Likert Scale (1 = Not at All, 5 = Extremely)

Question	Label
How severe do you think the novel coronavirus (COVID-19) will be for the general population?	Severity for German population
How harmful would it be for your health if you were to become infected COVID-19?	Harm to your health if infected
How concerned are you that you might become infected with COVID-19?	Concern you will be infected
How concerned are you that somebody you know might become infected with COVID-19?	Concern someone you know will be infected

Table B3

Items Assessing Government Response, Fatalities, and Compliance with Social Distancing Policies

Question	Answer options
**Government response (only Waves 3 and 4)**	
How understandable do you find the government's current policies and actions related to the COVID-19 pandemic?	5-Point Likert Scale (1 = Not at All, 5 = Completely)
How helpful do you find the government's current approach to the COVID-19 pandemic?	5-Point Likert Scale (1 = Not at All, 5 = Extremely)
Overall, how well do you think the governments of the following countries have handled the COVID-19 pandemic so far?	
(Wave 3: Australia, China, Germany, Italy, Singapore, South Korea, Spain, United States, United Kingdom)	5-Point Likert Scale (1 = Extremely, 5 = Not at All)
(Wave 4: Australia, China, Germany, Italy, Singapore, South Korea, Spain, United States, United Kingdom, Brazil, Russia, India, Sweden, France)	5-Point Likert Scale (1 = Extremely, 5 = Not at All)
**Fatalities (only Waves 2 and 3)**	
Please estimate the number of deaths caused by COVID-19 in each of the following countries (Australia, China, Germany, Italy, Singapore, South Korea, Spain, United States, United Kingdom)	Slider for numeric entry.
**Compliance (Wave 2 and 3)**	
In your opinion, what percentage of the population complies with the government's policy on social distancing?	Open numeric entry.
How much do you follow the government's policy on social distancing?	Multiple choice: I do not follow these guidelines at all; I follow these guidelines a little; I follow these guidelines somewhat; I follow these guidelines mostly, but not completely; I follow these guidelines completely; I go a little beyond what is required by government policy; I go a little beyond what government policy dictate; I go well beyond what government policy dictates; I am under complete quarantine and never leave home
**Compliance (AHA+L, Wave 4)**	
The federal government recommends the following combination of preventive measures to contain the COVID-19 pandemic, known as AHA + L rules: Keep your distance, observe hygiene, wear an everyday mask, and ventilate regularly. Have you heard about the AHA+L rules?	Yes/No/Partially
How much do you follow these AHA+L rules? (Keep distance (Keep at least 1.5 meters away from fellow humans); Observe hygiene (Cough and sneeze properly and wash or disinfect hands regularly); Wear everyday mask (If crowded: wear mouth/nose protection); Airing closed rooms regularly.)	5-Point Likert Scale (1 = Not at All, 5 = Completely)
What percentage of the German population do you think adheres fairly or completely to the AHA+L (distance, hygiene, everyday mask + ventilation) rules? Please estimate.	Open numeric entry.

Table B4
Scenarios Used in the Study (Translated from the German)

Scenario	Description	Wave
Severe	The COVID-19 pandemic has rapidly become a worldwide threat. Many experts agree that slowing the spread of the virus is essential to minimize the impact on the health care system and the economy, and to save many lives. The government might consider using mobile phone data to identify and contact those who may have come into contact with people with COVID-19. All people using a mobile phone would be included in the project, with no possibility of opting out. Data would be stored in an encrypted format on a secure server accessible only to the government, which may use the data to locate people who violate lockdown orders and fine or arrest them where necessary. Data would also be used to help shape the public health response and to contact people who might have been exposed to COVID-19. Individual quarantine orders could be made on the basis of this data.	1, 2
Mild	The COVID-19 pandemic has rapidly become a worldwide threat. Many experts agree that slowing the spread of the virus is essential to minimize the impact on the health care system and the economy, and to save many lives. The government could consider using mobile phone data to identify and contact those who may have come into contact with people with COVID-19. Only people who download a government app and agree to be tracked and contacted would be included in the project. The more people who download and use this app, the more effectively the government would be able to contain the spread of COVID-19. Data would be stored in an encrypted format on a secure server accessible only to the government. Data would only be used to contact those who might have been exposed to COVID-19.	1,2
Bluetooth	The COVID-19 pandemic has rapidly become a worldwide threat. Many experts agree that slowing the spread of the virus is essential to minimize the impact on the health care system and the economy, and to save many lives. Apple and Google have proposed adding a contact tracing capability to existing smartphones to inform people who have been exposed to others with COVID-19. This would help reduce community spread of COVID-19 by enabling people to voluntarily self-isolate. When two people are near each other, their phones would connect via Bluetooth. If a person is later identified as being infected, the people to whom they have been in close proximity are then notified without the government knowing who they are. The use of this contact tracing capability would be completely voluntary. People who are notified would not know the identity of the person who had tested positive.	2
Corona-Warn-App	The Corona-Warn-App app is designed to help detect and break infection chains at an early stage. Currently, local health authorities are trying to trace infection chains. With the app, this process can be automated and thus unfold much faster and more accurately. Users can be warned immediately if they have been in the vicinity of an infected person. The app was developed by Deutsche Telekom and SAP and published by the Robert Koch Institute. It records which smartphones have come in proximity to each other. To do this, smartphones with the app exchange randomly generated encryption keys via Bluetooth. The distance is estimated on the basis of the signal strength. If a user tests positive for COVID-19, they can share their test result in the app in order to inform users who have been in their vicinity. Infected users are explicitly asked whether they want to share their result for contact tracing. As an alternative to digital transmission, validation is available via a call center. Every 24 hours, the app checks whether the user has had contact with a person who has registered an infection on the app. The app does not evaluate any geodata and does not transmit any location information. The developers also assure that no personal data is sent or stored. The anonymized contact data is not stored centrally, but locally on the user's smartphone. The comparison of whether an infected person has been encountered is carried out locally on the smartphone. No data leaves the phone for matching, according to the developers. Only the anonymized list is stored centrally and regularly retrieved by the smartphones to identify possible infectious encounters.	3,4

Table B5

Items Assessing Acceptability of Tracking in the Three Hypothetical Scenarios and Downloads/Use of the Corona-Warn-App (CWA)

Scenario	Question	Label	Answer options
Severe	Is the use of cell phone data for location tracking acceptable in this scenario?	Acceptability of the scenario	Yes/No
Severe	Would your decision change if the government was required to delete the data and stop tracking after 6 months?	With follow up: delete data	Yes/No
Severe	Would your final decision change if there was an option to opt out of data collection?	With follow up: opt out	Yes/No
Mild	If, as depicted in this scenario, the government developed a tracking app to help reduce the spread of COVID-19, would you download and use it?	Acceptability of the scenario	Yes/No
Mild	Would your decision change if the government was required to delete the data and stop tracking after 6 months?	With follow up: delete data	Yes/No
Mild	Would your final decision change if data was only stored on your smartphone (not on government servers) and you had the option to make this data available if you tested positive for COVID-19?	With follow up: local data	Yes/No
Bluetooth	If, as depicted in this scenario, Apple and Google added a COVID-19 contact tracing capability to smartphones, would you use it?	Acceptability of the scenario	Yes/No
Bluetooth	Would your decision change if Apple and Google promised to delete all data and remove the contact tracing system after 6 months?	With follow up: delete data	Yes/No
CWA	Have you downloaded the Corona-Warn-App?	CWA downloads	Yes/No
CWA	Will you download the Corona-Warn-App in the future?	With follow up: future downloads	Yes/No
CWA	Is the Corona-Warn-App still installed on your phone?	–	Yes/No
CWA	Do you generally have Bluetooth switched on so the Corona-Warn-App can operate effectively?	–	Yes/No/Only when I leave the house/I don't know
CWA	Have you made any attempts to convince your friends and/or family to download the Corona-Warn-App?	–	Yes/No
CWA	Will you download the Corona-Warn-App in the future?	–	Yes/No
CWA	Do you think that the government should make the Corona-Warn-App mandatory?	–	Yes/No

Table B6

Items Assessing the Perceived Effectiveness and Risks of Hypothetical Tracking Apps in Different Scenarios (Waves 1 and 2) and of the Corona-Warn-App (CWA, Waves 3 and 4) on a 6-Point Likert Scale (1 = Not at All, 6 = Very).

Question	Label	Scenario
**Waves 1 and 2**		
How confident are you that the government app would reduce your likelihood of contracting COVID-19?	Reduce Likelihood to Contract	Severe, Mild, Bluetooth
How confident are you that the government app would reduce the spread of COVID-19?	Reduce Spread	Severe, Mild, Bluetooth
How confident are you that the government app would help you resume your normal activities more rapidly?	Return to Activity	Severe, Mild, Bluetooth
How easy is it for people to decline participation in the proposed project?	Ease of Declining Participation	Severe, Mild, Bluetooth
How serious is the risk of harm that could arise from the proposed project?	Risk of Harm	Severe, Mild, Bluetooth
How much do you agree that the government [Apple and Google for Bluetooth scenario] is collecting only necessary data?	Only Necessary Data Collection	Severe, Mild, Bluetooth
How sensitive is the data being collected in the proposed project?	How Sensitive Are Data	Severe, Mild, Bluetooth
How much do you trust the government to use the tracking data only to deal with the COVID-19 pandemic?	Trust: Data for Pandemic Only	Severe, Mild, Bluetooth
How much do you trust the government to be able to ensure the privacy of each individual?	Trust: Privacy Protection	Severe, Mild, Bluetooth
How secure is the data that would be collected for the proposed project from access by third parties ?	Trust: Security From 3rd Party	Severe, Mild, Bluetooth
To what extent do people have ongoing control of their data?	User Control Over Data	Severe, Mild, Bluetooth
How confident are you that other citizens like yourself would be able to download and effectively use the app?	Others' Ability to Download the App	Mild, Bluetooth
**Waves 3 and 4**		
How likely do you think it is that the Corona-Warn-App will reduce your risk of coming in contact with COVID-19?	Reduce Likelihood to Contract	CWA
Has the Corona-Warn-App already helped you to resume your normal activities?	Return to Activity Past	CWA
How confident are you that the Corona-Warn-App will help you to maintain your normal activities in the future course of the pandemic?	Return to Activity Future	CWA
To what extent do you think the Corona-Warn-App has already reduced the spread of COVID-19?	Reduce Spread Past	CWA
How confident are you that the Corona-Warn-App will reduce the spread of COVID-19?	Reduce Spread Future	CWA
How easy is it for people to decline participation in the Corona-Warn-App?	Ease of Declining Participation	CWA
How serious is the risk of harm that could arise from the proposed project?	Risk of Harm	CWA
How much do you trust that the Corona-Warn-App is collecting only necessary data?	Only Necessary Data Collection	CWA
How sensitive is the data being collected by the Corona-Warn-App?	How Sensitive Are Data	CWA
How much do you trust that the data will only be used to deal with the COVID-19 pandemic?	Trust: Data for Pandemic Only	
How much do you trust the app to be able to ensure the privacy of each individual?	Trust: Privacy Protection	CWA
How secure is the data collected by the Corona-Warn-App from access by third parties?	Trust:Security From 3rd Party	CWA
To what extent do people have ongoing control of their data? This includes control over how and when the data is collected, as well as access to view and delete the data after collection.	User Control Over Data	CWA
How confident are you that other citizens like yourself are able to download and effectively use the Corona-Warn-App?	Others' Ability to Download the App	CWA

Table B7
Reasons (Not) to Download the Corona-Warn-App (Multiple Responses Allowed)

Response Option	Label
**Why did you download the Corona-Warn-App? Please select all that apply.**	
To return to normal activities	To return to normal activities
To protect my health	To protect my health
To protect the health of others	To protect the health of others
To follow government recommendations	To follow government recommendations
To help the economy	To help the economy
Other reasons	
**Why won't you download the Corona-Warn-App? Please select all that apply.**	
I am concerned about privacy.	Privacy concerns
I don't trust the government.	Lack of gov trust
I am worried about battery usage on my phone.	Concerns: Battery usage
I don't think it will be effective.	Belief it is not effective
I am worried about normalizing government tracking.	Concerns: Normalizing gov tracking
I am concerned about civil liberties.	Concerns: Civil liberties
I don't own a smartphone.	Don't own a smartphone
My phone is too old to run the app.	Phone too old
I am concerned about others gaining access to my data.	Concerns: 3rd party access
I think that the virus doe not present a particular danger (Only Wave 4)	Belief virus is not dangerous
Other reasons (Only Wave 4)	Other reasons

Table B8
Items Assessing Worldviews

Name	Question	Scale
**Free market attitudes (Libertarianism)**	1. An economic system based on free markets unrestrained by government interference automatically works best to meet human needs. 2. The free market system may be efficient for resource allocation but it is limited in its capacity to promote social justice. (reverse coded) 3. The government should interfere with the lives of citizens as little as possible.	7-point Likert scale: 1 = strongly disagree, 7 = strongly agree
**Attitudes to science and technology**	1. Science and technology are making our lives healthier, easier, and more comfortable. 2. Because of science and technology, there will be more opportunities for the next generation.	7-point Likert scale: 1 = strongly disagree, 7 = strongly agree
**Belief in conspiracies (Wave 3)**	1. There are secret organizations that have great influence on political decisions. 2. Most people do not see how much of our lives are determined by plots hatched in secret. 3. There are certain political circles with secret agendas that are very influential. 4. I think that the various conspiracy theories circulating in the media are absolute nonsense. (reverse coded) 5. Secret organizations can manipulate people psychologically so that they do not notice how their life is being controlled by others. 6. Selfish interests have conspired to convince the public that COVID-19 is a major threat.	7-point Likert scale: 1 = strongly disagree, 7 = strongly agree
**Beliefs in conspiracies (Wave 4)**	1. COVID-19 does not really exist. It is a myth created by some influential people or institutions. 2. COVID-19 was created in a laboratory and deliberately released to achieve geopolitical or economic goals. 3. There is a link between 5G and the spread of COVID-19. 4. The severity of COVID-19 is overstated. The actual risk is not higher than that of a seasonal influenza. 5. The government exaggerates the seriousness of the pandemic in order to divert attention from other problems within Germany. 6. Wearing masks (mouth and nose protection) protects oneself and others from contracting a COVID-19 infection. (reverse coded) 7. COVID-19 was created by pharmaceutical companies to benefit from the need for a vaccine.	5-point Likert scale: 1 = undoubtedly false, 5 = undoubtedly true

.

Table B9
Items Assessing the General Acceptability of Privacy-Encroaching Measures During the Pandemic on a 4-Point Likert Scale (1 = Very Acceptable, 4 = Not Acceptable at All). Question: How Acceptable Is it For the Government to Take the Following Measures to Limit the Spread of the Virus During the COVID-19 Pandemic?

Item	Label
Provide access to the medical records of individuals.	Access to medical records
Track people's locations using their smartphone data.	Location tracking data
Enable temporary relaxation of data protection regulations.	Relaxation of data protection regulations
Collect data about personal contacts and interactions.	Data on contacts & interactions
Enforce people to use an app that notifies when those in quarantine leave the house.	Notification when people leave quarantine
Collect data on the infection and immunity status of citizens.	Data on infections & immunity status

Table B10
Variables and Items for Regression models

Variable	Items	Scale
**CWA download**	Have you downloaded the Corona-Warn-App?	Yes (1), No (0)
**Trust in CWA security**	1. How much do you trust the government to use the Corona-Warn-App data only to deal with the COVID-19 pandemic? 2. How much do you trust that the Corona-Warn-App can ensure the privacy of each individual who uses it? 3. How much do you trust that the Corona-Warn-App is collecting only necessary data? 4. How secure is the data collected by the Corona-Warn-App?	6-point Likert scale: 1 = not at all, 6 = very
**Perception of CWA effectiveness**	1. How confident are you that the Corona-Warn-App will help you to maintain your normal activities in the future course of the pandemic? 2. How confident are you that the Corona-Warn-App will reduce the spread of COVID-19? 3. How likely do you think it is that the Corona-Warn-App will reduce your risk of coming into contact with COVID-19?	6-point Likert scale: 1 = not at all, 6 = very
**CWA risk of harm perception**	How serious is the risk of harm that could arise from the Corona-Warn-App?	6-point Likert scale: 1 = not at all, 6 = very
**Acceptance of privacy limits**	How acceptable is it for the government to take the following measures to limit the spread of the virus during the COVID-19 pandemic? Combined measure for 6 items: See Appendix Table B9	4-point Likert scale: 1 = not acceptable at all, 4 = very acceptable
**Social media use & trust**	1. How often do you rely on the following media (social media) to keep you informed about the developments surrounding the COVID-19 pandemic? 2. How do you rate the following sources (social media)? 3. Do you think that the information on the COVID-19 pandemic is correct and trustworthy?	5-point Likert scale: 1 = never/not at all, 5 = always/completely
**Government announcements use & trust**	1.How often do you rely on the following media (government announcements) to keep you informed about developments surrounding the COVID-19 pandemic? 2. How do you rate the following sources(government announcements)? Do you think that the information on the COVID-19 pandemic is correct and trustworthy?	5-point Likert scale: 1 = never/not at all, 5 = always/completely
**COVID-19 risk perception**	Combined measure for 4 items: See Appendix Table B2	5-point Likert scale: 1 = not at all, 5 = extremely
**Approval of German pandemic response**	How well overall do you think the governments in the following countries have handled the COVID-19 pandemic? - Germany	5-point Likert scale: 1 = not at all, 5 = extremely
**Find government guidelines helpful**	How helpful are the government guidelines in deciding how to act in relation to the COVID-19 pandemic?	5-point Likert scale: 1 = not at all, 5 = extremely
**Attitudes to science and technology**	Combined measure for 2 items: See Appendix Table B8	7-point Likert scale: 1 = strongly disagree, 7 = strongly agree
**Free market attitudes (libertarianism)**	Combined measure for 3 items: See Appendix Table B8	7-point Likert scale: 1 = strongly disagree, 7 = strongly agree
**Belief in conspiracies (Wave 3)**	Combined measure for 6 items: See Appendix Table B8.	7-point Likert scale: 1 = strongly disagree, 7 = strongly agree
**Belief in conspiracies (Wave 4)**	Combined measure for 7 items: See Appendix Table B8	5-point Likert scale: 1 = undoubtedly false, 5 = undoubtedly true
**Gender (male)**	What gender do you identify with? (Note: "Other" category was excluded from regression analyses due to its small sample size, see Table 1)	Male (1), Female (0)
**Education**	Please indicate your highest level of education.	"Low" = None, *Hauptschule*, *Realschule*; "Medium" = *Abitur*, "High" = University
**Age**	How old are you?	Free numeric text entry
